# Smoke and Spike: Benzo[a]pyrene Enhances SARS‐CoV‐2 Infection by Boosting NR4A2‐Induced ACE2 and TMPRSS2 Expression

**DOI:** 10.1002/advs.202300834

**Published:** 2023-07-10

**Authors:** Wenbin Liu, Yue Zhao, Junyan Fan, Jiaying Shen, Hailin Tang, Wanda Tang, Di Wu, Weijin Huang, Yibo Ding, Peng Qiao, Jiansheng Lin, Zishuai Li, Qianqian Li, Qianqian Cui, Yan Liu, Yifan Chen, Rui Pu, Xue Han, Jianhua Yin, Xiaojie Tan, Guangwen Cao

**Affiliations:** ^1^ Key Laboratory of Biosafety Defense Ministry of Education Shanghai 200433 China; ^2^ Shanghai Key Laboratory of Medical Biodefense Shanghai 200433 China; ^3^ Department of Epidemiology Second Military Medical University Shanghai 200433 China; ^4^ Shanghai East Hospital Key Laboratory of Arrhythmias Ministry of Education Tongji University School of Medicine Tongji University Shanghai 200331 China; ^5^ Department of Microbiology Second Military Medical University Shanghai 200433 China; ^6^ Seven Plus Technology Company Limited Tianjin 300301 China; ^7^ Institute for Biological Product Control National Institutes for Food and Drug Control WHO Collaborating Center for Standardization and Evaluation of Biologicals NHC Key Laboratory of Research on Quality and Standardization of Biotech Products and NMPA Key Laboratory for Quality Research and Evaluation of Biological Products Beijing 102629 China; ^8^ Department of Infectious Disease Control Center for Disease Control and Prevention of Yangpu District Shanghai 200090 China; ^9^ Department of Epidemiology School of Medicine Jinan University Guangzhou China

**Keywords:** angiotensin‐converting enzyme 2, cigarettes, coronavirus disease 2019, NR4A2, transmembrane protease serine 2

## Abstract

Cigarette smoke aggravates severe acute respiratory syndrome coronavirus 2 (SARS‐CoV‐2) infection. However, the underlying mechanisms remain unclear. Here, they show that benzo[a]pyrene in cigarette smoke extract facilitates SARS‐CoV‐2 infection via upregulating angiotensin‐converting enzyme 2 (ACE2) and transmembrane protease serine 2 (TMPRSS2). Benzo[a]pyrene *trans*‐activates the promoters of *ACE2* and *TMPRSS2* by upregulating nuclear receptor subfamily 4 A number 2 (NR4A2) and promoting its binding of NR4A2 to their promoters, which is independent of functional genetic polymorphisms in *ACE2* and *TMPRSS2*. Benzo[a]pyrene increases the susceptibility of lung epithelial cells to SARS‐CoV‐2 pseudoviruses and facilitates the infection of authentic Omicron BA.5 in primary human alveolar type II cells, lung organoids, and lung and testis of hamsters. Increased expression of Nr4a2, Ace2, and Tmprss2, as well as decreased methylation of CpG islands at the Nr4a2 promoter are observed in aged mice compared to their younger counterparts. NR4A2 knockdown or interferon‐λ2/λ3 stimulation downregulates the expression of NR4A2, ACE2, and TMPRSS2, thereby inhibiting the infection. In conclusion, benzo[a]pyrene enhances SARS‐CoV‐2 infection by boosting NR4A2‐induced ACE2 and TMPRSS2 expression. This study elucidates the mechanisms underlying the detrimental effects of cigarette smoking on SARS‐CoV‐2 infection and provides prophylactic options for coronavirus disease 2019, particularly for the elderly population.

## Graphical abstract

Benzo[a]pyrene is an active cigarette smoke ingredient that enhances severe acute respiratory syndrome coronavirus 2 infection by boosting nuclear receptor subfamily 4 A number 2 (NR4A2)‐induced expression of viral receptors, angiotensin‐converting enzyme 2 and transmembrane protease serine 2. NR4A2 is epigenetically up‐regulated in aged individuals and silenced by interferon‐λ3, which provides prophylactic options for coronavirus disease 2019 in the elderly.

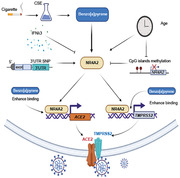

## 1 Introduction

Severe acute respiratory syndrome coronavirus 2 (SARS‐CoV‐2), the causative agent of coronavirus disease 2019 (COVID‐19), had claimed 6 656 601 lives globally as of December 23, 2022.^[^ ^1^ ^]^ The ongoing spread and evolution of SARS‐CoV‐2 have resulted in the emergence of new variants, including Omicron BA.4 and BA.5, which exhibit increased immune evasion capability and enhanced replication efficacy, demanding changes in epidemic prevention and control strategies.^[^ ^2^, ^3^ ^]^

Despite its evolution, the viral entry mechanism of SARS‐CoV‐2 relies on angiotensin‐converting enzyme 2 (ACE2) and transmembrane protease serine 2 (TMPRSS2). The spike (S) protein of SARS‐CoV‐2 consists of two functional domains, S1 and S2, responsible for receptor binding and fusion with the cellular membrane, respectively. The S1 component comprises an N‐terminal domain and a receptor‐binding domain (RBD). Similar to SARS‐CoV‐1, the RBD of the S protein facilitates viral entry by binding to ACE2 during SARS‐CoV‐2 infection.^[^ ^4^, ^5^ ^]^ The RBD of the S protein is one of the most commonly targeted regions for the development of novel vaccines and detection technologies.^[^ ^6^, ^7^, ^8^ ^]^ TMPRSS2, an activator of ACE2, plays a critical role in priming the S protein for complex formation with ACE2 and facilitating SARS‐CoV‐2 entry into host cells.^[^ ^9^ ^]^ As SARS‐CoV‐2 rapidly evolves, the S protein exhibits increasingly higher binding affinity for ACE2.^[^ ^2^ ^]^ Although the S protein of Omicron BA.1 is less dependent on TMPRSS2 compared to the ancestral strain, the S protein of BA.5 reacquires its dependency on TMPRSS2.^[^ ^10^, ^11^, ^12^ ^]^ Immunohistochemistry (IHC) and single‐cell sequencing studies have demonstrated the co‐localization and co‐expression of ACE2 and TMPRSS2 in different organs and tissues.^[^ ^9^, ^13^ ^]^ High expression levels of ACE2 and TMPRSS2 facilitate viral infection and replication, resulting in a higher viral load that correlates with the severity of COVID‐19.^[^ ^14^ ^]^ Understanding the regulatory mechanism of ACE2 and TMPRSS2 is crucial for improving the prophylaxis and treatment of SARS‐CoV‐2 and other related viruses.^[^ ^15^ ^]^

Epidemiological studies have identified cigarette smoking as a risk factor for SARS‐CoV‐2 infection, severe illness, and adverse outcomes. A study in the UK involving 421 469 participants reported a higher risk of SARS‐CoV‐2 infection associated with daily cigarette consumption, with an odds ratio of 2.51 (95% confidence interval [CI]:1.20–5.24).^[^ ^16^ ^]^ Another study with 954 patients demonstrated that individuals with a smoking history had a significantly increased risk of severe illnesses compared to nonsmokers, with an OR of 5.5 (95% CI:3.1–9.9).^[^ ^17^ ^]^ The underlying biological mechanisms by which the constituents of tobacco products influence the immune and inflammatory responses to SARS‐CoV‐2 infection need to be elucidated.^[^ ^18^ ^]^ Furthermore, recent studies have demonstrated that cigarette smoking significantly upregulates ACE2 and TMPRSS2 expression in various cell lines.^[^ ^19^, ^20^, ^21^ ^]^ Cigarette contains a complex mixture of harmful constituents including nicotine, benzo[a]pyrene (BaP), tobacco tar, and carbon monoxide. However, the specific harmful constituents of cigarette smoke that upregulate the two SARS‐CoV‐2 receptors and their underlying mechanisms remain unclear. Besides cigarette smoking, other factors, such as exposure to poor air quality, older age, comorbidities, lower socioeconomic status, and race (Hispanics, Blacks, and Indigenous people), in the USA have been reported to increase the risk of COVID‐19 mortality.^[^ ^22^, ^23^, ^24^, ^25^, ^26^, ^27^ ^]^ Disparities in exposure, susceptibility to infection, capacity to treat infection, and hospitalization may contribute to COVID‐19 mortality rates.^[^ ^28^ ^]^ Factors affecting the susceptibility to and severity of SARS‐CoV‐2 infection, as well as the mechanisms by which external and internal factors upregulate ACE2 and TMPRSS2, remain unclear. In this study, we identified BaP as the major active ingredient of cigarette smoke extract (CSE) that facilitates SARS‐CoV‐2 infection, and we found that nuclear receptor subfamily 4 group A member 2 (NR4A2) is a critical transcription factor of ACE2 and TMPRSS2. Furthermore, we also demonstrated the impact of aging, immunoregulatory cytokines, and genetic polymorphisms on the expression of ACE2 and TMPRSS2.

## 2 Results

### 2.1 Cigarette Smoke Upregulates the Expression of ACE2 and TMPRSS2 by Increasing Their Promoter Activity

**Figure 1:**
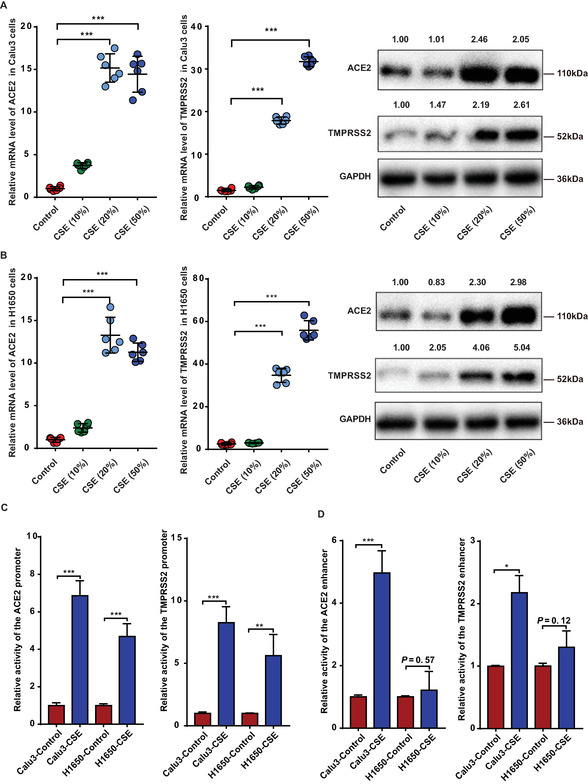
Effects of cigarette smoke extract on mRNA, protein levels, promoter activities, and enhancer activities of ACE2 and TMPRSS2. Calu3 and H1650 cells for RT‐qPCR and western blot were stimulated with CSE at different concentrations (0%, 10%, 20%, and 50%) for 12 h (A,B). Cells for luciferase assays were stimulated with CSE at 0% and 20% for 12 h (C,D). All experiments were repeated three times with six replicates and one of the representative results is shown here. The activities of the promoters and enhancers with wild‐type genotypes of SNPs were displayed here. A,B) mRNA and protein levels of ACE2 and TMPRSS2 in Calu3 (A) and H1650 cells (B). RT‐qPCR and western blot analyses were normalized using GAPHD as an internal control. C,D) Luciferase assays to evaluate the activities of the ACE2 and TMPRSS2 promoters (C) and enhancers (D). Relative mRNA, protein levels, and luciferase activity are presented as a ratio of the CSE group to the control group (0% CSE). *n* = 3 for all experiments. CSE: cigarette smoke extract. The activities of the promoters and enhancers with wild‐type genotypes of SNPs are displayed here. Student's *t‐*test, *p < 0.05, **p < 0.005, ***p < 0.0005. Error bars represent standard deviation.

The RNA and protein levels of ACE2 and TMPRSS2 were assessed in lung cancer cell lines 12 h after stimulation with different concentrations (10%, 20%, and 50%) of CSE. Concentrations of 20% and 50% CSE significantly increased mRNA and protein levels of ACE2 and TMPRSS2 in Calu3 and H1650 cells, respectively (**Figure** **1**A,B). Luciferase assays revealed that 20% CSE significantly increased the activities of *ACE2* and *TMPRSS2* promoters (−2000 to −1 bp) in both Calu3 and H1650 cells (Figure 1C). Furthermore, in Calu3 cells, CSE significantly increased the activities of *ACE2* and *TMPRSS2* enhancers (−5000 to −2000 bp) with no significant effect in H1650 cells (Figure 1D).

To validate our findings, we analyzed three transcriptome datasets from the Gene Expression Omnibus (GSE994, GSE8987, and GSE10718) and the Cancer Genome Atlas lung adenocarcinoma (LUAD) cohorts. The GSE994 and LUAD datasets provided gene expression data for bronchial epithelial cells and tumor tissues from lung cancer patients with different histories of smoking, respectively. The CSE8987 dataset provided information regarding buccal epithelial cells from healthy nonsmokers and current smokers. The GSE10718 dataset contained data on bronchial epithelial cell lines exposed to air and smoke. The expression of *ACE2* was significantly higher in current smokers compared to former smokers and nonsmokers, and significantly higher in individuals with a smoking history of more than 15 years compared to those with a smoking history of 15 years or less, as shown in the LUAD dataset (Figure S1A, Supporting Information). The effects of cigarette smoke exposure and duration on the increased expression of *TMPRSS2* were evident in all datasets (GSE994, LUAD, GSE8987, and GSE10718) (Figure S1B, Supporting Information).

### 2.2 CSE Impact on the Transcription of *ACE2* and *TMPRSS2* is Not Influenced by Functional Genetic Polymorphisms

Functional single nucleotide polymorphisms (SNPs) were selected using RegulomeDB software (http://www.regulomedb.org/) and HaploReg software (http://archive.broadinstitute.org/mammals/haploreg). The selected SNPs included rs112312217 (the *ACE2* enhancer, −4340 bp, A>G), rs113208650 (the *ACE2* promoter, −1035 bp, G>A), rs9981570 (the *TMPRSS2* enhancer, −3164 bp, A>G), rs8128074 (the *TMPRSS2* promoter, −1485 bp, A>G), and rs12481984 (the *TMPRSS2* promoter, −887 bp, A>G). The minor allele frequency of each SNP was >5% in at least one human race, according to the 1000 Genomes Project dataset (http://www.1000genomes.org).

**Figure 2:**
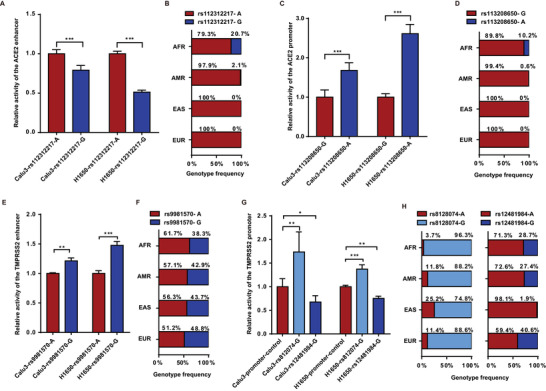
Effects of single nucleotide polymorphisms on the activities of the *ACE2* and *TMPRSS2* transcriptional regulatory sequences. A) Activity of the *ACE2* enhancer with reference genotype (A) and variant genotype (G) at rs112312217. B) Frequency of rs112312217 genotypes across different human races. C) Activity of the *ACE2* promoter with reference genotype (A) and variant genotype (G) rs113208650. D) Frequency of rs113208650 genotypes across different human races. E) Activity of the *TMPRSS2* enhancer with reference genotype (A) and variant genotype (G) at rs9981570. F) Frequency of rs9981570 genotypes across different human races. G) Activity of the *TMPRSS2* promoter with reference genotype (A) and variant genotype (G) at rs12481984 and rs812074. H) Frequency of rs12481984 and rs812074 genotypes in human races. Activity of each construct was normalized to the construct containing the reference allele (rs112312217‐A, rs113208650‐G, rs9981570‐A, or rs12481984A‐rs812074‐A). The relative luciferase activity was calculated by comparing the activity of each promoter/enhancer construct to the reference genotype. *n* = 3 for all experiments. Student's *t*‐test, ^*^ *p* < 0.05, ^**^ *p* < 0.005, ^***^ *p* < 0.0005. Error bars represent standard deviation. AFR, African population; AMR, American population; EAS, East Asian population; EUR, European population.

To confirm the functions of these five SNPs, luciferase assays were conducted in Calu3 and H1650 cells. The activity of the *ACE2* enhancer with rs112312217‐G was significantly lower than that with rs112312217‐A (**Figure** **2**A). Notably, rs112312217‐G was predominantly found in the African population, with lower prevalence in East Asian and European populations (Figure 2B). Conversely, the activity of the *ACE2* promoter with rs113208650‐A was significantly higher compared to rs113208650‐G (Figure 2C). The rs113208650‐A allele was mainly observed in the African population (Figure 2D). The activity of the *TMPRSS2* enhancer with rs9981570‐G was significantly higher compared to rs9981570‐A (Figure 2E), and the rs9981570‐G had an approximate frequency of 40% in African, American, East Asian, and European populations (Figure 2F). Additionally, the activity of the *TMPRSS2* promoter with rs12481984‐G and rs812074‐A was significantly higher than with rs12481984‐A and rs812074‐A, while significantly lower with rs12481984‐A and rs812074‐G compared to rs12481984‐A and rs812074‐A (Figure 2G). The rs8128074‐G was more frequent in the African population, whereas the rs12481984‐G was less frequent in the East Asian population (Figure 2H,I). However, the genotypes of these SNPs did not influence the overall effect of CSE on the transcriptions of *ACE2* and *TMPRSS2* (Figure S2, Supporting Information).

### 2.3 BaP is the Active CSE Ingredient Able to Upregulate the Expression of ACE2 and TMPRSS2

Among the harmful constituents of CSE, including nicotine, carbon monoxide, and tar, BaP, and nicotine were specifically investigated in subsequent studies. Carbon monoxide is difficult to dissolve in water, and tar is a complex mixture of compounds, therefore, were not investigated.

**Figure 3:**
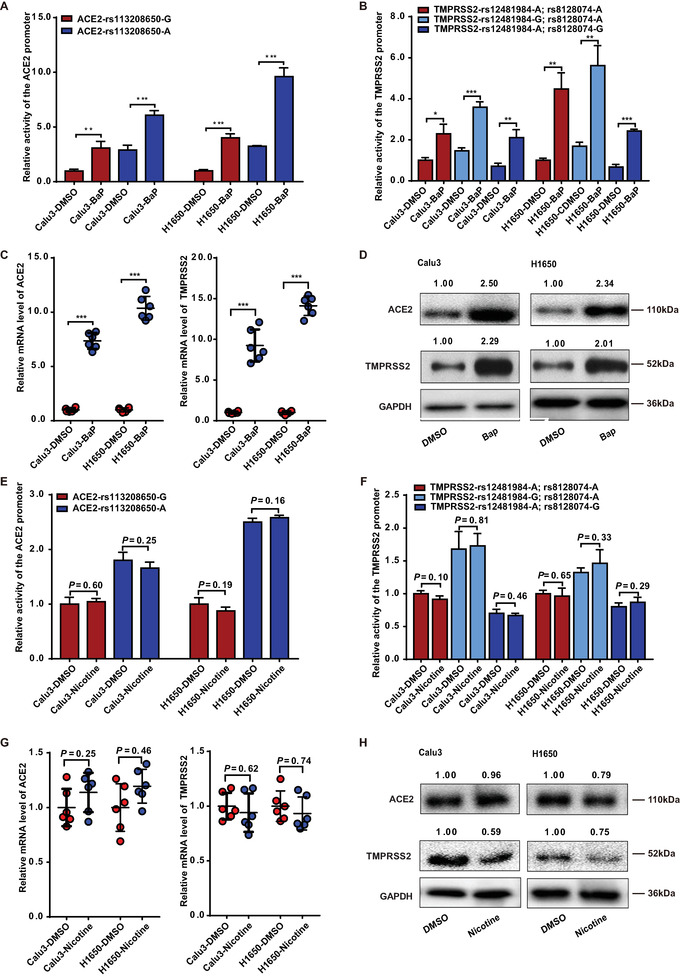
Benzo[a]pyrene, rather than nicotine, is the active ingredient of CSE that upregulates ACE2 and TMPRSS2. A,B) Luciferase assays to evaluate the activity of the *ACE2* promoter (A) and *TMPRSS2* promoter (B) in cells stimulated with benzo[a]pyrene (BaP) (15 µm, 12 h). C,D) qPCR and western blot analyses to evaluate the expression of ACE2 and TMPRSS2 in cells stimulated with BaP (15 µm, 12 h). E,F) Luciferase assays to evaluate the activities of the *ACE2* and *TMPRSS2* promoters in cells stimulated with nicotine (100 µm, 12 h). G,H) qPCR and western blot analyses to evaluate the expression of ACE2 and TMPRSS2 in cells stimulated with nicotine (100 µm, 12 h). Cells in the control group were treated with 0.1% DMSO. RT‐qPCR and western blot analyses were normalized using GAPHD as an internal control. Relative mRNA, protein levels, and luciferase activity are presented as a ratio of the target group (0% BaP or Nicotine group) compared to the DMSO group. *n* = 3 for all experiments. BaP: benzo[a]pyrene. Student's *t*‐test, *p < 0.05, **p < 0.005, ***p < 0.0005. Error bars represent standard deviation.

Luciferase assays demonstrated that BaP (15 µm, 12 h) significantly upregulated the activities of the *ACE2* and *TMPRSS2* promoters. These effects were unrelated to the rs113208650, rs12481984, and rs8128074 genotypes (**Figure** **3**A,B). Moreover, BaP treatment promoted the increase of ACE2 and TMPRSS2 mRNA and protein levels (Figure 3C,D). On the other hand, nicotine (12 h, 100 µm) did not significantly affect the promoter activities and mRNA levels of ACE2 and TMPRSS2 (Figure 3E–G). Interestingly, nicotine significantly downregulated the protein levels of TMPRSS2 but not of ACE2 (Figure 3H). We further validated the effects of BaP in primary human alveolar type II (AT II) cells. Stimulation with BaP for 12, 24, and 48 h significantly upregulated ACE2 and TMPRSS2 expression in AT II cells (Figure S3A, Supporting Information). Additionally, BaP significantly promoted the transcription of other potential SARS‐CoV‐2 receptors including asialoglycoprotein receptor, syndecan 4, and kringle containing transmembrane protein 1 KREMEN1 (Figure S3B, Supporting Information).

### 2.4 NR4A2 is an Essential Transcription Factor for the Expression of ACE2 and TMPRSS2

The promoter regions (−2000 to −1 bp) of *ACE2* and *TMPRSS2* were used to predict their putative transcription factors. By cross‐validating data from the JASPAR and RegulomeDB databases, we identified 48 and 39 putative transcriptions that bind to *ACE2* and *TMPRSS2* promoters, respectively (Table S1, Supporting Information). To investigate the relationship between cigarette smoking and these transcription factors, we analyzed the GSE994, GSE10718, GSE8987, and LUAD datasets. Notably, NR4A2 and cAMP‐responsive element modulator (CREM) were significantly upregulated, while snail family transcriptional repressor 2 (SNAI2) was downregulated in samples exposed to CSE, and these effects were observed across multiple databases (Table S2, Supporting Information).

**Figure 4:**
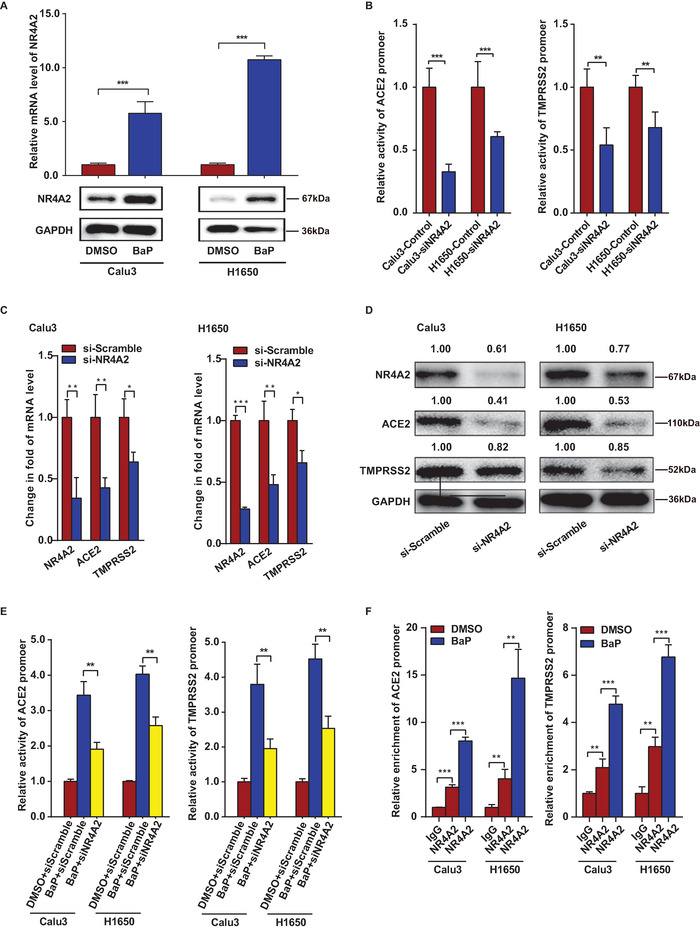
BaP promotes the expression of ACE2 and TMPRSS2 via NR4A2. Cells were transfected with siNR4A2 or siScramble for 24 h before analysis. For luciferase assays, after transfection, cells were then stimulated with BaP for 12 h. A) qRT‐PCR and western blot analyses to evaluate NR4A2 in cells stimulated with BaP. B) Luciferase assays to examine the activities of the *ACE2* and *TMPRSS2* promoters in cells with NR4A2 knockdown. C,D) qRT‐PCR and western blot analyses assessing the expression of ACE2 and TMPRSS2 in cells with NR4A2 knockdown. E) Knockdown of NR4A2 attenuated the effects of BaP on the activities of *ACE2* and *TMPRSS2* promoters. F) ChIP‐qPCR analysis showing the enrichment of the ACE2 and TMPRSS2 promoters in genomic DNA binding to NR4A2. Relative enrichment is presented as a ratio of the qPCR value for the NR4A2 group to that of the IgG group. *n* = 3 for all experiments. DMSO: cells cultured with DMSO (0.1%); BaP: cells cultured with BaP (15 µm, 12 h); IgG: DNA pulled down by IgG antibody; NR4A2: DNA pulled down by NR4A2 antibody. Student's *t*‐test, *p < 0.05, **p < 0.005, ***p < 0.0005. Error bars represent the standard deviation.

Our experimental results revealed that CSE significantly increased the transcription of NR4A2 in both Calu3 and H1650 cells. However, the effects of CSE on the expression of CREM and SNAI2 were inconsistent in different cell lines (Figure S4, Supporting Information). Based on these findings, NR4A2 was selected for further investigation. Moreover, BaP significantly increased NR4A2 expression in both Calu3 and H1650 cells (**Figure** **4**A). Subsequently, the knockdown of NR4A2 using siRNA significantly decreased the promoter activities, mRNA levels, and protein levels of ACE2 and TMPRSS2 (Figure 4B–D). NR4A2 knockdown significantly attenuated the BaP‐induced increase in *ACE2* and *TMPRSS2* promoter activity (Figure 4E). The effect of NR4A2 on the activities of *ACE2* and *TMPRSS2* promoters was independent of the genotypes rs113208650, rs12481984, and rs812074 (Figure S5, Supporting Information).

Our chromatin immunoprecipitation (ChIP)‐qPCR experiments confirmed the interaction between NR4A2 and the *ACE2* and *TMPRSS2* promoter regions in both Calu3 and H1650 cells, demonstrating enhanced binding capacity in the presence of BaP (Figure 4F). Additionally, the ChIP‐qPCR product was verified through Sanger sequencing (Figure S6, Supporting Information). Moreover, an assay for transposase‐accessible chromatin sequencing (ATAC‐seq) revealed that BaP significantly increased the chromatin accessibility of the NR4A2 promoter region, while it had no significant effects on the accessibility of the *ACE2* and *TMPRSS2* promoters in Calu3 cells (Table S3, Supporting Information). Therefore, BaP upregulates the activities of *ACE2* and *TMPRSS2* promoters by enhancing the expression and binding capacity of NR4A2.

### 2.5 NR4A2 is Involved in Age‐, Inflammation‐, and Genetic Polymorphism‐Mediated Regulation of ACE2 and TMPRSS2 Expression

**Figure 5:**
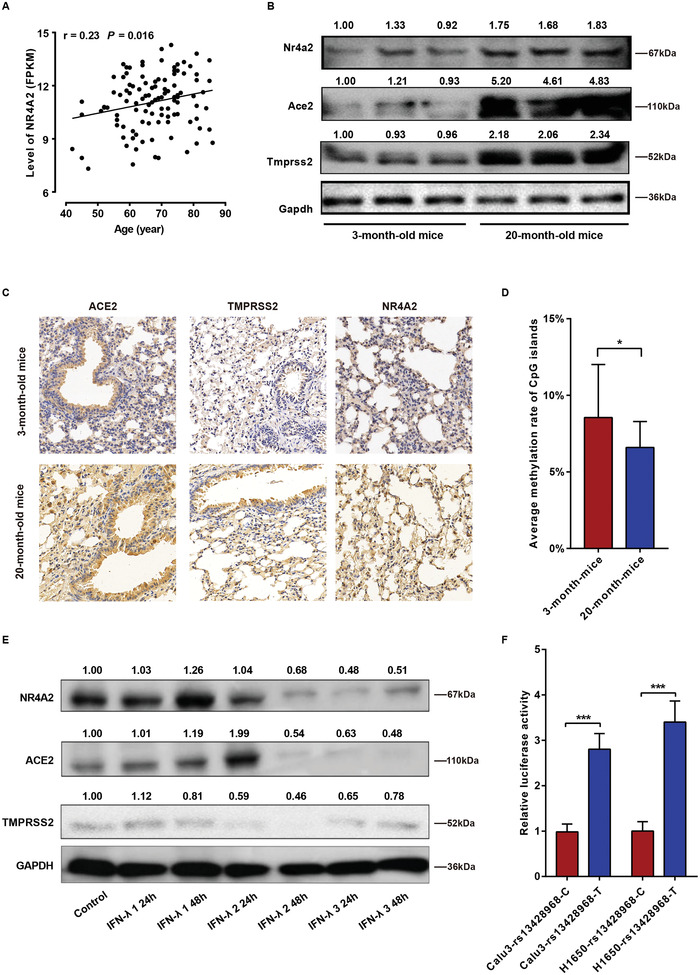
The involvement of NR4A2 in age‐, inflammation‐, and genetic polymorphism‐mediated regulation of ACE2 and TMPRSS2. A) Positive correlation between age and the mRNA levels of NR4A2 in lung tissues, as determined using Pearson's correlation with the data of normal tissues from patients of TCGA LUCD and LUSC cohorts. *n* = 74. B,C) The representative images of western blot and immunohistochemistry showing upregulation of Nr4a2 expression in lung tissues of 20‐month‐old mice compared to 3‐month‐old mice. Scale bar, 50 µm. *n* = 3 per group. D) Methylation rate of CpG islands within the promoter of *Nr4a2* in mouse lung tissues. *n* = 3 per group. E) Representative image of western blot test showing protein levels of NR4A2, ACE2, and TMPRSS2 in H1650 cell line stimulated with IFN‐λ. *n* = 3 per group. F) Effect of genotypes at rs13428968 within the 3′UTR region on the stability of NR4A2 mRNA. *n* = 3 per group. Student's *t*‐test, *p < 0.05, **p < 0.005, ***p < 0.0005. Error bars represent standard deviation.

Analyzing gene expression data for non‐cancerous lung tissues in the TCGA cohorts of LUAD and lung squamous cell carcinoma, we observed significant positive correlations between age and expression level of *NR4A2* (**Figure** **5**A). Furthermore, we analyzed methylation data from both cohorts and found an inverse correlation between age and the methylation levels at 93.10% (27 of 29) of CpG sites within the transcriptional regulation region (+500 bp to −1.2 × 10^4^ bp) of *NR4A2*. Notably, four CpG sites showed a statistically significant negative correlation between age and methylation (Table S4, Supporting Information).

To further investigate the age‐related regulation of Nr4a2, we examined the expression of Nr4a2, Ace2, and Tmprss2 in the lung tissues of mice of different ages. Interestingly, the expression levels of these genes were significantly higher in the lung tissues of 20‐month‐old mice compared to the 3‐month‐old mice (Figure 5B,C and Figure S7, Supporting Information). To assess the methylation rates of eight CpG islands within the *Nr4a2* promoter region, lung tissues of C57BL/6J mice (NC_000068.8, GRCm39, Chr2:57006420‐57006637) were subjected to sodium bisulfite pyrosequencing. The average DNA methylation rates of the eight CpGs were lower in 20‐month‐old mice than in 3‐month‐old mice (Figure 5D and Figure S8, Supporting Information).

We conducted in vitro experiments to investigate the effects of nine inflammatory factors on the expression of NR4A2, ACE2, and TMPRSS2. However, no stable and consistent results of qPCR and western blot assays were observed in Calu3 and H1650 cells stimulated with interferon‐α (IFN‐α), tumor necrosis factor‐α (TNF‐α), interleukin‐1α (IL‐1α), IL‐1β, IL‐4, and IL‐6 (Figure S9, Supporting Information). Interestingly, stimulation with IFN‐λ2 (48 h) and IFN‐λ3 (24 and 48 h) significantly decreased the protein levels of NR4A2, ACE2, and TMPRSS2, whereas IFN‐λ1 had no significant effect on the expression of these three genes (Figure 5E).

We identified a female COVID‐19 patient who lived with her husband and daughter, both free of infection. All three family members were genotyped for SNPs using microarray analysis. No differences in the genotypes of SNPs within the *ACE2* and *TMPRSS2* promoter regions were detected. However, this COVID‐19 patient carried the TT genotype at rs13428968, an SNP within 3′UTR of *NR4A2*, while her husband and her daughter carried the rs13428968‐TC genotype. Luciferase assays indicated that the mRNA of *NR4A2* with rs13428968‐T at the 3′ untranslated region (UTR) was more stable than that with rs13428968‐C (Figure 5F).

### 2.6 The Influence of CSE, BaP, NR4A2, and IFN‐λ3 on the Susceptibility to SARS‐CoV‐2 Infection

**Figure 6:**
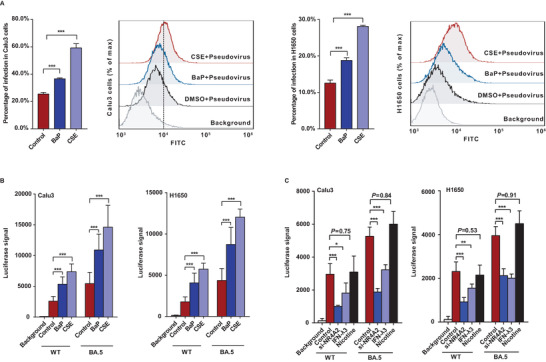
Effects of CSE and BaP on cell susceptibility to infection with pseudoviruses expressing the spike protein of SARS‐CoV‐2. A) Percentage of GFP‐positive Calu3 and H1650 cells assessed by flow cytometry following infection with lentivirus‐based pseudovirus expressing the wild‐type spike protein of SARS‐CoV‐2 (MOI = 2; 48 hpi). Cells were incubated with 0.1% DMSO, 20% CSE, and 15 µM BaP for 12 h. Background signal value was detected with cells without stimulation and infection. B,C) Luciferase assays evaluating the infection of VSV‐based pseudovirus. Background: luciferase signal in cells neither stimulated nor infected with pseudovirus. Cells were incubated with BaP (15 µm), CSE (20%), nicotine (100 µm), and IFN‐λ3 (50 ng mL^−1^) before infection with pseudovirus. Cells were transfected with siRNA against NR4A2 for 48 h. WT: cells infected with VSV‐based pseudovirus of wild‐type SARS‐CoV‐2. BA.5: cells infected with VSV‐based pseudovirus of Omicron BA.5. *n* = 3 for all experiments. Student's *t*‐test, *p < 0.05, **p < 0.005, ***p < 0.0005. Error bars represent standard deviation.

To evaluate the effects of CSE and BaP on cellular susceptibility to viral infection, we used a lentivirus‐based pseudovirus expressing the green fluorescent protein (GFP)‐fused S protein of SARS‐CoV‐2. The level of pseudovirus infection was evaluated by measuring the percentage of GFP‐positive cells using flow cytometry. As shown in **Figure** **6**A, the percentage of infected cells significantly increased in the cells treated with CSE and BaP for 12 h. To confirm these findings, we used vesicular stomatitis virus (VSV) pseudoviruses carrying wild‐type SARS‐CoV‐2 and Omicron BA.5 with luciferase expression (Figure 6B). BaP significantly inhibited the infection of VSV‐based pseudoviruses carrying the VSV G glycoprotein instead of the SARS‐CoV‐2 S protein (Figure S10A, Supporting Information). The anti‐spike antibody effectively inhibited pseudoviral infection and attenuated the positive effects of BaP on pseudoviral infection (Figure S10B, Supporting Information). These results suggest that BaP specifically promotes infection by targeting the SARS‐CoV‐2 S protein rather than other surface proteins of VSV. Knockdown of NR4A2 and stimulation with IFN‐λ3 significantly inhibited the infection of wild‐type SARS‐CoV‐2 and Omicron BA.5 pseudoviruses, whereas nicotine did not significantly affect infection (Figure 6C).

**Figure 7:**
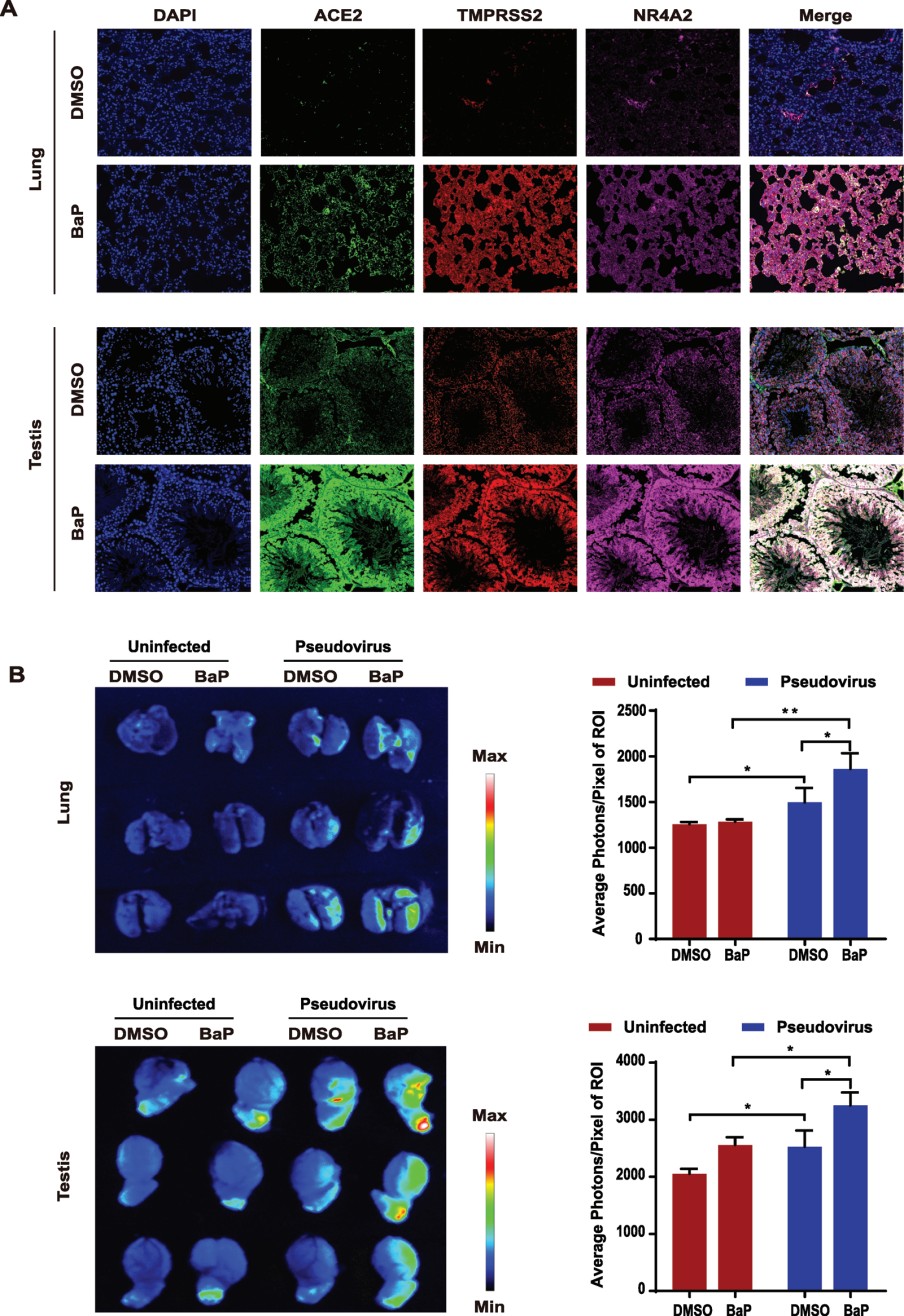
BaP upregulates the expression of Nr4a2, Ace2, and Tmprss2 proteins and facilitates infection of SARS‐CoV‐2 pseudovirus in lung and testis tissues of hamsters. A) Multiplex immunofluorescence staining images of lung and testis tissues from healthy golden Syrian hamster. Scale bar, 50 µm. B) Ex vivo live images and quantitative analysis of image data for lung and testis tissues of healthy golden Syrian hamsters. *n* = 3 per group. Hamsters were injected with BaP (12 5 mg kg^−1^) into the left pleural cavity and scrotum. 3 weeks later, hamsters were injected with lentivirus‐based Omicron pseudovirus into the left pleural cavity and scrotum (each 250 µL, 2 × 10^7^ U mL^−1^). GFP fluorescence was detected 2 days later. The flux of GFP fluorescence in the region of interest was measured to evaluate the infection level of pseudovirus. Student's *t*‐test, *p < 0.05, **p < 0.005, ***p < 0.0005. Error bars represent standard deviation.

We also examined the impact of BaP in vivo using hamsters that received intrapleural and intrascrotal injections of BaP (125 mg kg^−1^) and were subsequently infected with Omicron BA.5 pseudoviruses with the same approach 3 weeks later. The expression levels of Nr4a2, Ace2, and Tmprss2 proteins, as well as the infection level of Omicron BA.5 pseudovirus, were significantly higher in the lung and testis tissues of BaP‐treated hamsters compared to dimethyl sulfoxide (DMSO)‐control hamsters (**Figure** **7** and Figure S11, Supporting Information). Pseudovirus infection was repeatable in the lungs of mice (Figure S12, Supporting Information), indicating that BaP significantly enhances susceptibility to SARS‐CoV‐2 infection.

**Figure 8:**
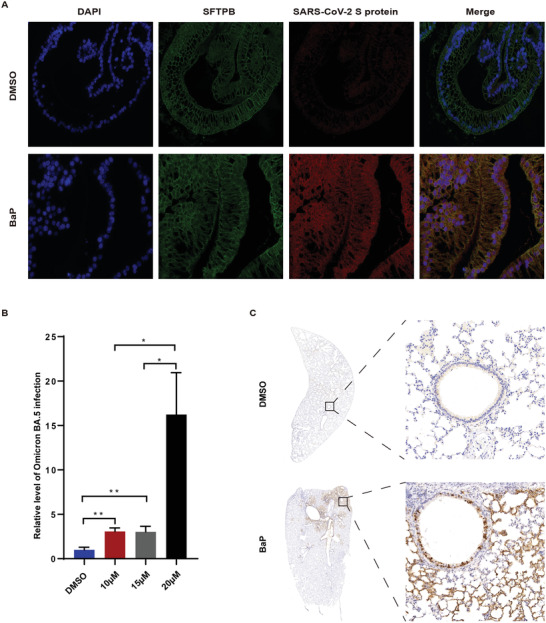
BaP promotes the infection of authentic strains of Omicron in vivo and in vitro. A) Representative multiplex immunofluorescence staining images of iPSC‐derived lung organoids. Scale bar, 50 µm. *n* = 3 per group. The iPSC‐derived lung organoids, containing AT II epithelial cells (marked by SFTPB protein), were stimulated with BaP (15 µm) for 12 h and then infected with Omicron BA.5 at MOI of 1 for 24 h. B. B) Infection assays in primary human AT II cells. Primary human AT II cells were stimulated with BaP at concentrations of 10, 15, and 20 µm for 12 h and then infected with Omicron BA.5 at MOI of 1 for 24 h. The viral load was evaluated by detecting SARS‐CoV‐2 N gene copies, normalized to GAPDH. The relative level of Omicron BA.5 infection is presented as a ratio of the BaP group to the DMSO group. *n* = 6 per group. C) Representative IHC image of lung tissues of hamsters injected with BaP (125 mg kg^−1^) or equal value of DMSO into left pleural cavity and intranasally infected with 1 × 10^5^ PFU of Omicron BA.5 3 weeks later. IHC was conducted at 48 hpi. Scale bar, 50 µm. *n* = 3 per group. *p < 0.05, **p < 0.005, ***p < 0.0005. Error bars represent standard deviation.

Furthermore, we verified the positive effect of BaP on SARS‐CoV‐2 infection using an authentic strain of Omicron BA.5, both in vitro and in vivo. Furthermore, induced pluripotent stem cell (iPSC)‐derived lung organoids and primary human AT II cells were stimulated with BaP for 12 h and subsequently infected with Omicron BA.5 at an MOI of 1 for 24 h. Multiple immunofluorescence staining revealed that BaP significantly promoted Omicron BA.5 infection in AT II cells within iPSC‐derived lung organoids (**Figure** **8**A). The level of Omicron BA.5 was 15.2‐fold higher in primary AT II cells treated with BaP (20 µm) compared to those treated with DMSO (Figure 8B). Hamsters received intrapleural injections of BaP (125 mg kg^−1^) and were intranasally infected with 1 × 10^5^ plaque‐forming units (PFU) of Omicron BA.5 3 weeks after BaP treatment. IHC analysis confirmed that chronic stimulation with BaP significantly promoted Omicron BA.5 infection in lung tissues (Figure 8C).

## 3 Discussion

In this study, we investigated the impact of CSE on the expression of ACE2 and TMPRSS2 and the susceptibility of SARS‐CoV‐2 infection to lung epithelial cells. Consistent with previous epidemiological studies and in vitro cell experiments, CSE upregulated ACE2 and TMPRSS2 expression, increasing the cells' vulnerability to viral infection.^[^ ^12^, ^13^, ^14^, ^15^, ^16^, ^17^ ^]^ TCGA data analysis demonstrated increased levels of ACE2 and TMPRSS2 in smokers, which decreased after smoking cessation (Figure S1, Supporting Information). We identified BaP as the active ingredient in CSE responsible for trans‐activating the expression of ACE2 and TMPRSS2, while nicotine had no significant effect on their transcription or susceptibility to SARS‐CoV‐2 infection. A previous study had shown that cigarette smoking, rather than e‐cigarette smoking, upregulated ACE2 expression, suggesting that ACE2 upregulation in smokers was not due to nicotine.^[^ ^29^ ^]^ BaP, a main polycyclic aromatic hydrocarbon, is a well‐known oncogenic compound, present in cigarette smoke, vehicle exhaust gases, smoked and cooked food products, surface water, soil, sediments, and crops.^[^ ^30^, ^31^ ^]^ BaP is also a significant component of air pollutants.^[^ ^32^ ^]^ Epidemiological studies have linked exposure to poor air quality and polluted materials with increased severity and mortality of COVID‐19.^[^ ^22^, ^26^, ^27^, ^33^ ^]^ However, the specific effects of BaP on transcriptional regulation and viral infections have been scarcely investigated. A study conducted in Poland revealed that exposure to elevated levels of particulate matter <2.5 µm and BaP was associated with a higher risk of early respiratory symptoms of COVID‐19 and a hyper‐inflammatory state, increasing COVID‐19 mortality.^[^ ^34^ ^]^ This study sheds light on the mechanism through which BaP exposure increases the risk of COVID‐19 mortality. We found that BaP facilitates SARS‐CoV‐2 infection by upregulating the transcription and translation of ACE2 and TMPRSS2. Therefore, overexposure to cigarette smoke and BaP‐contaminated food, living necessities, and environments might increase the severity and mortality of COVID‐19 by increasing the abundance of SARS‐CoV‐2 receptors in pneumonocytes. Moreover, individuals with lower socioeconomic, who are more likely to be exposed to BaP‐containing pollution and contaminated living necessities, may face an increased risk of COVID‐19 mortality.

Furthermore, we uncovered that BaP upregulates the expression of SARS‐CoV‐2 receptors by increasing the mRNA and protein levels of the transcription factor NR4A2 and promoting the binding of NR4A2 to the *ACE2* and *TMPRSS2* promoters. NR4A2, also known as Nurr1, is an orphan nuclear receptor involved in immunology, neurodegenerative pathology, and cancer.^[^ ^35^, ^36^, ^37^, ^38^ ^]^ NR4A2 is significantly activated during the differentiation of human type II pneumocytes, the major targets of SARS‐CoV‐2.^[^ ^13^, ^39^ ^]^ The expression of NR4A2 positively correlates with aging in human lung tissue expression datasets, and older mice exhibit higher Nr4a2 expression than younger mice. The expression levels of Ace2 and Tmprss2 were also found to be higher in aged mice, possibly due to Nr4a2 trans‐regulation. The upregulation of NR4A2 in elderly individuals may be partially attributed to decreased methylation of the *Nr4a2* promoter. However, the mechanism through which BaP activates the expression of NR4A2 requires further investigation. A previous study has suggested that BaP induces somatic mutations in the p53 coding gene, which could inhibit p53 expression.^[^ ^40^ ^]^ p53 downregulates NR4A2 via increasing miR‐34a, a miRNA targeting 3′UTR of NR4A2,^[^ ^41^ ^]^ therefore, BaP may increase NR4A2 expression through inhibition of p53 expression. Although BaP exhibits a mean half‐life of 46.5 ± 58.2 h,^[^ ^30^ ^]^ BaP‐induced genetic injury, including p53 mutations, accumulates with age. BaP can also regulate gene expression by recruiting methyltransferases to gene promoters.^[^ ^42^ ^]^ Therefore, BaP likely affects the expression of NR4A2 through epigenetic mechanisms. This indicates, at least partially, that both BaP and older age contribute to the severity and mortality of SARS‐CoV‐2 infection by activating NR4A2.

Functional SNPs of ACE2 and TMPRSS2 have been shown to significantly affect SARS‐CoV‐2 infection.^[^ ^43^, ^44^, ^45^ ^]^ In this study, we identified that rs13428968‐T within the 3′UTR region of NR4A2 contributes to the stability of NR4A2 mRNA, potentially affecting the risk of SARS‐CoV‐2 infection. We also identified functional SNPs that could impact the activity of *ACE2* and *TMPRSS2* promoters or enhancers. However, these SNPs may not have a uniform effect across different races. For instance, the frequencies of ACE2 promoter SNP rs113208650‐A and ACE2 enhancer SNP rs112312217‐G were higher in the African population compared to other races, but these two SNPs exhibit opposite effects. While rs113208650‐A increases ACE2 promoter activity, rs112312217‐G decreases ACE2 enhancer activity. Moreover, the functional SNPs only induced a modest increase (1.6–2.8‐fold) in the activities of the ACE2 or TMPRSS2 promoters, whereas exposure to CSE resulted in a 4.2–8.1‐fold increase in promoter activity independent of SNPs. Hence, external exposure, such as CSE, has a more pronounced effect than genetic factors.

Our study also provides insights into therapeutic agents for controlling COVID‐19. Antitumor drugs like camptothecin and celecoxib, as well as novel compounds such as KU0171309, inhibit the transcription and function of NR4A2.^[^ ^46^, ^47^ ^]^ These drugs may be promising options for the prevention and treatment of SARS‐CoV‐2 and other coronaviruses by targeting ACE2 and TMPRSS2. NR4A2 also acts as an upstream regulator of several inflammatory factors.^[^ ^35^ ^]^ However, the feedback effects of inflammatory factors on the expression of NR4A2 have not been extensively studied. Notably, IFN‐λ members play crucial roles in the defense against SARS‐CoV‐2 infection. The lack of early IFN‐λ responses contributes to the severe transformation of SARS‐CoV‐2 infection.^[^ ^48^ ^]^ Here, we uncovered a novel mechanism for the anti‐viral function of IFN‐λ, as IFN‐λ2 and IFN‐λ3 significantly downregulate the expression of NR4A2, ACE2, and TMPRSS2, with IFN‐λ3 showing a more stable effect and significantly reducing susceptibility to SARS‐CoV‐2 infection. Thus, IFN‐λ3 could be a choice for prophylaxis and treatment of COVID‐19.

Despite the valuable findings of our study, several limitations should be acknowledged. We focused on the effects of BaP on AT II cells, lung cancer cells, and lung organoids. ACE2 expression and regulation showed significant heterogeneity across different cell types, which may explain why some studies did not observe an association between ACE2 and TMPRSS2 expression and cigarette smoking in patients with advanced cell lung cancer or the bronchial epithelial cell line.^[^ ^49^, ^50^ ^]^ Therefore, further investigation is required to explore the effects of BaP on different cell types and tissues. Additionally, our study primarily investigated the mechanisms of transcriptional regulation of ACE2 and TMPRSS2. While nicotine downregulated the protein levels of TMPRSS2 without affecting its mRNA, the effects of IFN‐α, TNF‐α, IL‐1α, IL‐1β, IL‐4, and IL‐6 on the mRNA and protein levels of ACE2 and TMPRSS2 were inconsistent. It is crucial to consider post‐translational regulation as an influencer of ACE2 and TMPRSS2, which warrants further investigation. Finally, our findings on the inhibitory effects of IFN‐λ2 and IFN‐λ3 on NR4A2, ACE2, and TMPRSS2, highlight their potent anti‐SARS‐CoV‐2 effect. Previous studies have demonstrated the age‐dependent effectiveness of IFN‐λ against infections of human coronavirus, including SARS‐CoV‐2, with low levels of inflammation.^[^ ^51^, ^52^, ^53^, ^54^ ^]^ Additional research is required to elucidate the mechanisms through which BaP and IFN‐λ regulate the expression of NR4A2, ACE2, and TMPRSS2 in nasal and bronchial epithelial cells.

## 4 Conclusion

BaP is the major active ingredient in CSE that facilitates the expression of SARS‐CoV‐2 receptors and promotes SARS‐CoV‐2 infection by upregulating the transcription factor NR4A2. NR4A2 is involved in the age‐dependent and IFN‐λ3‐mediated regulation of ACE2 and TMPRSS2. Notably, IFN‐λ emerges as a promising therapeutic target for counteracting the NR4A2 upregulation induced by BaP, thereby exhibiting promising anti‐COVID‐19 activity in the BaP‐exposed populations.

## 5 Experimental Section

### Cell Lines and Organoids

Calu3, H1650, African green monkey kidney cell line Vero E6, and HEK293T cells were purchased from the cell bank of the Chinese Academy of Sciences (Shanghai, China). Primary human AT II was purchased from Procell Co. Ltd (Wuhan, China) and cultured with F12 medium (CORNING, NY, USA) supplemented with 10% FBS, and 1% penicillin/streptomycin at 37 °C and 5% CO_2_. Prior to experiments, all cell lines were authenticated by genotyping of short tandem repeats using Biowing Biotechnology (Shanghai, China). Additionally, all cell cultures were tested for mycoplasma contamination every 3 months. Human iPSC‐derived lung organoids were generated by Seven Plus Limited Company (Tianjin, China) as previously described.^[^ ^55^ ^]^ Further details on cell culture can be found in the Supporting Information.

### CSE

Freshly prepared CSE was used for each experiment, following a previously described protocol with minor modifications.^[^ ^56^ ^]^ Briefly, cigarette smoke (Hademen, Shandong, China) was dissolved in a 25 mL cell culture medium using a negative‐pressure suction instrument (−9 kPa, Shanyifeng, Shanghai, China). The obtained CSE solution was filtered (0.22 µm, Corning, Corning, NY, USA) to remove insoluble particles, resulting in a 100% CSE solution. The concentration of 10% CSE used in experiments approximately corresponded to the effect of exposure to cigarette smoking equivalent to two packs per day.^[^ ^56^ ^]^

### Gene Expression Analysis

mRNAs and protein levels were determined using real‐time quantitative PCR (RT‐qPCR) and western blotting, respectively. Cells were stimulated with different concentrations of CSE (10%, 20%, and 50%), 15 µm BaP (Luone, Beijing, China), 100 µm nicotine (Fusheng, Beijing, China), or 50 ng mL^−1^ of each inflammatory factor (IFN‐α, TNF‐α, IL‐1α, IL‐1β, IL‐4, IL‐6, IFN‐λ1, IFN‐λ2, and IFN‐λ3 (Sigma‐Aldrich, Santa Clara, CA, USA)) for 12 h and then harvested for RT‐qPCR and western blot analysis. Glyceraldehyde‐3‐phosphate dehydrogenase (GAPDH) was used as an internal control for qRT‐PCR and western blot. The change in the mRNA levels was calculated relative to that of the control group. The signal intensity of the western blot bands was quantified using ImageJ software (version 1.51, https://imagej.nih.gov/ij/) and normalized to GAPDH. The relative signal intensity of the western blot bands was calculated as the ratio of the target group to the control group. All experiments were repeated thrice with at least three replicates. Further details of RT‐qPCR and western blotting, including information on the primers and antibodies, are available in the Supporting Information.

### Plasmids

DNA fragments of the *ACE2* enhancer region (500 bp flanking rs112312217), *ACE2* promoter region (−2000 to −1 bp containing rs113208650), *TMPRSS2* enhancer region (500 bp flanking rs9981570), and *TMPRSS2* promoter region (−2000 to −1 bp containing rs8128074 and rs12481984) were synthesized and sequenced by Obio Technology (Shanghai, China). The correct promoter and enhancer sequences were cloned into promoter‐free luciferase reporter vectors (pGL4.10‐basic vector, Promega, Madison, WI, USA) and enhancer‐free luciferase reporter vectors (pGL4.26‐basic vector, Promega), respectively. These constructs were validated by DNA sequencing.

### Luciferase Assays

Cells were transfected with a firefly luciferase reporter vector and Renilla luciferase control reporter vector (Promega) using Lipofectamine LTX, according to the manufacturer's instructions (Invitrogen, Carlsbad, CA, USA). After 24 h, luciferase activity was measured using a dual‐luciferase reporter assay system (Promega, WI, USA). Firefly luciferase activity was normalized to Renilla luciferase activity. To investigate the effects of CSE, nicotine, BaP, and inflammatory factors on the activity of transcriptional elements, cells were treated with the compounds or inflammatory factors for 12 h prior to the luciferase assay. Cells in the control group were incubated with 0.1% DMSO (Sigma, Santa Clara, CA, USA). To investigate the effects of transcription factors on the activity of transcriptional elements, cells were transfected with small interfering RNA (siRNA) targeting NR4A2 for 24 h prior to stimulation and luciferase assays. siRNAs targeting NR4A2 and the scrambled sequence (siNR4A2, siScramble) were synthesized by RiboBio (Guangzhou, China). All luciferase assays were repeated thrice with at least three replicates. The siRNA sequences are listed in the Supporting Information.

### Chromatin Immunoprecipitation Assay

ChIP assays were performed using the SimpleChIP Plus Enzymatic Chromatin IP Kit (Cell Signaling Technology, USA) according to the manufacturer's instructions. Chromatin–protein complexes were immunoprecipitated using an antibody against NR4A2 (Cell Signaling Technology). IgG was used as an experimental negative control (Cell Signaling Technology). ChIP‐quantitative PCR (ChIP‐qPCR) was performed using the cellular genomic DNA bound to NR4A2. Primers for ChIP‐qPCR were specifically designed to amplify *ACE2* and *TMPRSS2* promoters (details provided in the Supporting Information). The enrichment percentage was calculated using the following formula: Enrichment percentage = 2% × 2^(C[T] of input − C[T] of sample)^, where C[T] represents the threshold cycle for qPCR. The fold‐change in the enrichment of site occupancy was determined by comparing the DNA values of the NR4A2 group with those of the IgG control group (three replicates per group)_._ The amplified ChIP‐qPCR products were verified by Sanger sequencing which was performed by Shanghai Sangon Biotech Co., Ltd.

### Bisulfite Pyrosequencing Assay

DNA was extracted from mouse lung tissues using a TIANGEN genomic DNA extraction kit, according to the manufacturer's instructions (Beijing, China). Bisulfite conversion was conducted using the EZ DNA Methylation‐Gold kit (Zymo Research, CA, USA) following the manufacturer's instructions. Pyrophosphate sequencing analysis was performed using a PyroMark Q96 ID instrument (Qiagen, Hilden, Germany) and PyroMark Gold Q96 reagent (Qiagen).

### Animal

All animal studies were conducted according to the animal welfare protocol approved by the Ethics Committee of the Second Military Medical University. Male C57BL/6 mice and golden Syrian hamsters (8‐week‐old) were purchased from Weitong Lihua Experimental Animals Co., LTD (Beijing, China).

### Pseudovirus Infection Assay

Lentivirus‐based pseudoviruses expressing GFP were purchased from Yeasen Biotech (1 × 10^7^ TU mL^−1^, Shanghai, China). VSV‐based pseudoviruses were constructed as previously described.^[^ ^57^ ^]^ The full‐length S genes from wild‐type SARS‐CoV‐2 (Wuhan‐Hu‐1 strain, GenBank: MN908947) and the Omicron variant (GISAID: EPI_ISL_6590782.2) were inserted into pcDNA3.1. Subsequently, 293T cells were transfected with pcDNA3.1‐based constructs and simultaneously infected with G* G‐VSV expressing firefly luciferase (Kerafast, Boston, MA, USA) to produce pseudoviruses.^[^ ^58^, ^59^ ^]^ Viral titers were determined in 293T cells using threefold serial dilutions. Cells were then infected with pseudovirus at a multiplicity of infection (MOI) of 2.0 and collected 48 h after transduction. A VSV‐based pseudovirus carrying the VSV G gene was used as the control. Lentivirus‐ and VSV‐based pseudovirus infection efficiency was evaluated by flow cytometry and luciferase assays, respectively.

Hamsters and mice were randomly divided into BaP and control groups (*n* = 3 per group). BaP (125 mg kg^−1^) was dissolved in DMSO (Sigma, 300 µL for hamsters and 60 µL for mice) and injected into the left pleural space in both animal models and intrascrotally injected into each hamster testicle. The animals in the control group received equal doses of DMSO. 3 weeks later, hamsters received an intrapleural injection (250 µL) and intrascrotal injection (250 µL) of lentivirus‐based pseudovirus (2 × 10^7^ U mL^−1^), while mice only received an intrapleural injection (100 µL). 2 days after infection, lung tissues and testicles were collected for western blotting and fluorescence imaging. The fluorescent signals of ex vivo organs were evaluated using an IVIS imaging system (IVIS Spectrum, Xenogen, CA, USA).

### Infection Assay with Authentic Omicron BA.5

The Omicron BA.5 strain was isolated from a laboratory‐confirmed COVID‐19 patient and passaged in Vero E6 cells as previously described.^[^ ^60^ ^]^ Virus working stocks were propagated and titrated in Vero E6 cells in the presence of TPCK‐treated trypsin at a concentration of 2 µg mL^−1^. The resulting virus was stored at −80 °C. All experiments involving the authentic Omicron BA.5 strain were performed in the Biosafety Level 3 facility at the Second Military Medical University.

Human primary AT II cells were stimulated with BaP at a concentration of 15 µm for 12 h and infected with Omicron BA.5 at an MOI of 1 for 24 h. The AT II cells were harvested using the TRIzol reagent for qRT‐PCR, following the manufacturer's instructions. Viral load was determined by quantifying SARS‐CoV‐2 N gene copies, which were normalized to the GAPDH. The relative level of Omicron BA.5 infection was expressed as the ratio of the BaP group to the DMSO group.

The iPSC‐derived lung organoids were constructed at the liquid–liquid interface and exposed to BaP prior to infection with Omicron BA.5 under air–liquid interphase conditions. The lung organoids were stimulated with BaP at 15 µm for 12 h and infected with Omicron BA.5 at an MOI of 1 for 24 h. Following the infection, the lung organoids were fixed in a 10% formalin solution for subsequent multiplex immunofluorescence staining.

In the animal model, hamsters were randomly divided into two groups, with three animals per group. Hamsters were injected with BaP (125 mg kg^−1^) or an equal amount of DMSO in the left pleural cavity. 3 weeks later, the hamsters were intranasally infected with 1 × 10^5^ PFU of Omicron BA.5. After 48 h post‐infection (hpi), hamsters were euthanized, and their lungs collected for IHC analyses. The antibodies used for immunofluorescence are described in detail in the Supporting Information.

### Human Peripheral Blood Samples and SNP Array Analysis

Peripheral blood samples were obtained from a female COVID‐19 patient and her two family members after the patient was clinically cured and discharged. The three participants provided written informed consent, and the study protocol was in accordance with the ethical guidelines of the Declaration of Helsinki and was approved by the Ethics Committee of the Second Military Medical University. Genomic DNA was extracted from the blood samples and subjected to SNP array analysis using the Infinium Asian Screening Array‐24 v1.0 (Illumina, San Diego, CA, USA) according to the manufacturer's protocol.

### ATAC‐Seq Analysis

Calu3 cells were divided into two groups and treated with BaP (15 µm) and DMSO (0.1%) for 12 h, respectively (three replicates per group). Nuclei preparation and bulk ATAC‐seq library construction were conducted using the Hyperactive ATAC‐Seq Library Prep Kit (Vazyme, Nanjing, China) according to the manufacturer's instructions. Sequencing was performed using a HiSeq platform (Illumina, San Diego, CA, USA). The resulting reads were aligned and peaks were called using the GRCh38 Human Genome Reference with the Seurat software.

### Application of Public Data

Three microarray datasets (GSE994, GSE8987, and GSE10718) were obtained from the GEO database.^[^ ^61^, ^62^, ^63^ ^]^ The GSE994 and GSE8987 datasets provided gene expression data for epithelial cells from the intrapulmonary airway and buccal mucosa, respectively, collected from individuals with different smoking histories. The GSE10718 dataset provided gene expression data of NHBE cells exposed to air and smoke. To further complement the analysis, gene expression and methylation datasets of TCGA LUAD and LUSC cohorts were acquired from the UCSC Xena website (https://xenabrowser.net/). The datasets included relevant information such as age, smoking history, and gene expression. The expression data were presented as RNA‐seq by expectation‐maximum values, which underwent quantile‐normalized and log_2_ transformation.

### Statistical Analysis

All experiments were independently repeated thrice with a minimum of three replicates, and representative results are presented in the text. Quantification of RT‐qPCR data and the intensity of western blot bands were normalized to the reference gene. Luciferase assay data were normalized to the Renilla luciferase activity. Relative mRNA levels, protein levels, and luciferase activity are presented as the ratio of the target group to the control group. Data are presented as mean values and standard deviations. Statistical differences in continuous variables were determined using the Student's *t*‐test. For non‐normally distributed data, including expression data obtained from the GEO and TCGA datasets, results are presented as medians with interquartile ranges. Differences in non‐normally distributed values were determined using the Mann–Whitney U test. Pearson's correlation analysis was employed to evaluate the relationships between gene expression levels. All statistical tests were two‐sided and conducted using SPSS (version 16.0; SPSS, Chicago, IL, USA). A *p* < 0.05 was considered statistically significant.

## Conflict of Interest

The authors declare no conflict of interest.

## Author Contributions

W.L., Y.Z., J.F., J.S., H.T., and W.T. contributed equally to this work. Study concept and design: G.C.; Experiments: Y.Z., J.F., J.S., W.L., H.T., W.T., D.W., W.H., Y.D., Y.L., Z.L., Y.C., Q.L., Q.C., P.Q., and X.H.; Bioinformatics analysis and statistical analysis: W.L., J.L.; Technical supports: R.P., J.Y., X.T.; Interpretation of the data and drafting of the manuscript: W.L. and G.C.; Revision of the manuscript and study supervision: G.C.

## Supporting information

Supporting Information

## Data Availability

The data that support the findings of this study are available from the corresponding author upon reasonable request.
