# Supplementary material for: Smoke and Spike: Benzo[a]pyrene Enhances SARS‐CoV‐2 Infection by Boosting NR4A2‐Induced ACE2 and TMPRSS2 Expression
Source: Adv Sci (Weinh). 2023 Jul 10;10(26):2300834. doi: 10.1002/advs.202300834 (PMC10502855; doi:10.1002/advs.202300834)
Supplement: Supplementary file 1 — Supporting Information [file ADVS-10-2300834-s001.pdf]

ADVANCED SCIENCE
Open Access

Supporting Information

for *Adv. Sci.*, DOI 10.1002/advs.202300834

Smoke and Spike: Benzo[a]pyrene Enhances SARS-CoV-2 Infection by Boosting NR4A2-Induced ACE2 and TMPRSS2 Expression

*Wenbin Liu, Yue Zhao, Junyan Fan, Jiaying Shen, Hailin Tang, Wanda Tang, Di Wu, Weijin Huang, Yibo Ding, Peng Qiao, Jiansheng Lin, Zishuai Li, Qianqian Li, Qianqian Cui, Yan Liu, Yifan Chen, Rui Pu, Xue Han, Jianhua Yin, Xiaojie Tan and Guangwen Cao**

Supplementary Materials

Cell culture

Calu3 cells, H1650 cells, African green monkey kidney cell line Vero E6 cells, and HEK293T cells were purchased from the cell bank of the Chinese Academy of Sciences (Shanghai, China). Calu3, African green monkey kidney cell line Vero E6 cells, and HEK293T cell lines were cultured in Dulbecco's Modified Eagle Medium (DMEM, GIBCO, Paisley, UK). The H1650 cell line was cultured in RPMI-1640 Medium (GIBCO). Cell culture medium was supplemented with 10% fetal bovine serum (GIBCO), 100 U/ml of penicillin, and 100 μg/ml streptomycin (Invitrogen, Carlsbad, CA). Cells were grown in an incubator at 37°C, 5% CO_2 condition.

Real-time quantitative PCR (RT-qPCR) and Western blot

Total RNA was isolated, reversely transcribed into cDNA, and used as a template for RT-qPCR as previously described.[1] RT-qPCR was performed using SYBR Green reagents (Takara, Japan). The sequences of primers are listed in Table S5. The program for the quantitative PCR is as follows: Step 1, 95°C for 3 min; Step 2, 45 cycles of 95°C for 10s, 60°C for 10s; Step 3, 72°C for 25s. Gene expression was relatively quantified using *GAPDH* as an internal control. Each qRT-PCR assay was conducted in triplicate. Protein was extracted, quantified, and subjected to Western blot according to standard protocols as previously described.[2] The primary antibodies used in this study including western blot tests, immunohistochemistry, and multiplex immunofluorescence are listed in Table S6. All of these primary antibodies react with mouse, rat, and human proteins. They were used for testing the protein levels in mouse tissues, hamster tissues, and human cell lines. GAPDH was used as the loading control for cellular proteins.

Knockdown of putative transcription factors

Gene knockdown was conducted with gene-specific siRNA. The sequence of siRNA targeting *NR4A2*: siNR4A2, 5'- CCACTACGCACATGATCGA-3'. The cells were transfected with siRNA by using lipofectamine LTX (Invitrogen). The efficiency of knockdown was confirmed by using RT-qPCR.

Primers for ChIP-qPCR

ACE2 promoter: forward primer, AGTGACAGGAGAGGTAAGG; reverse primer, AACAATCTGCTGAGCCAAT. TMPRSS2 promoter: forward primer, CTCA GTG GCCACACGGAAGAT; reverse primer, ATTTCTGCATGCCCCTCCACA.

Reference:

1. Li, X., Tan, X., Yu, Y., Chen, H., Chang, W., Hou, J., Xu, D., Ma, L., Cao, G. (2011). D9S168 microsatellite alteration predicts a poor prognosis in patients with clear cell renal cell carcinoma and correlates with the down-regulation of protein tyrosine phosphatase receptor delta. Cancer *117*, 4201–4211. https://doi.org/10.1002/cncr.26028
2. an, X., Wang, Y., Han, Y., Chang, W., Su, T., Hou, J., Xu, D., Yu, Y., Ma, W., Thompson, T. C., Cao, G. (2013). Genetic variation in the GSTM3 promoter confer risk and prognosis of renal cell carcinoma by reducing gene expression. Br. J. Cancer, *109*, 3105–3115. https://doi.org/10.1038/bjc.2013.669

Figure S1

A

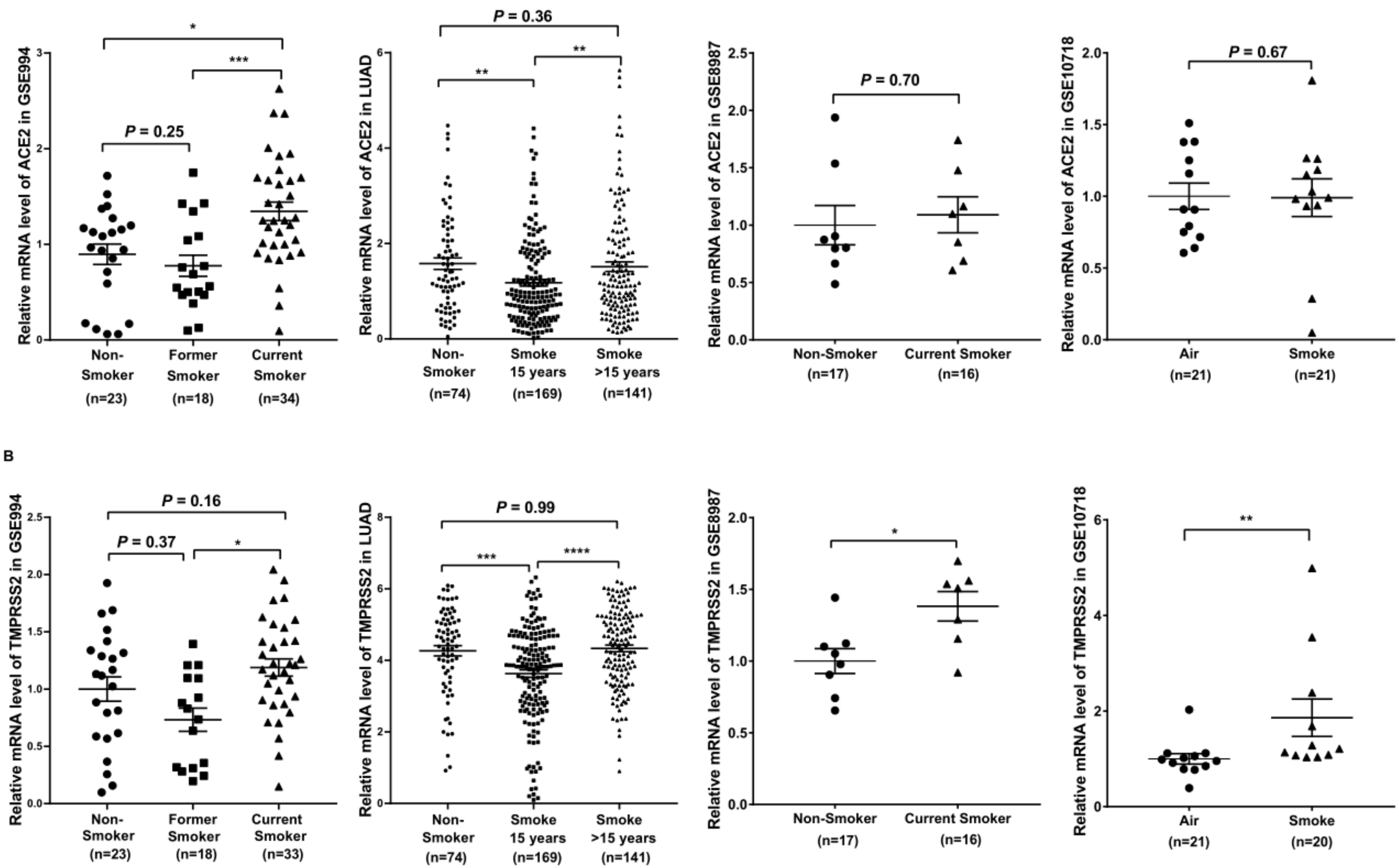

Fi

Figure S1. Effects of cigarette smoke exposure on the mRNA levels of ACE2 and TMPRSS2

(A) The effect of cigarette smoke exposure on the mRNA level of ACE2 was evaluated with the use of public databases regarding GSE994, TCGA LUAD, GSE8987, and GSE10718. (B) The effect of cigarette smoke exposure on the mRNA level of TMPRSS2 was evaluated with public databases regarding GSE994, TCGA LUAD, GSE8987, and GSE10718. Mann-Whitney test, $^{*}P$<0.05, $^{**}P$<0.005, $^{***}P$<0.0005. Error bars represent standard deviation.

Figure S2

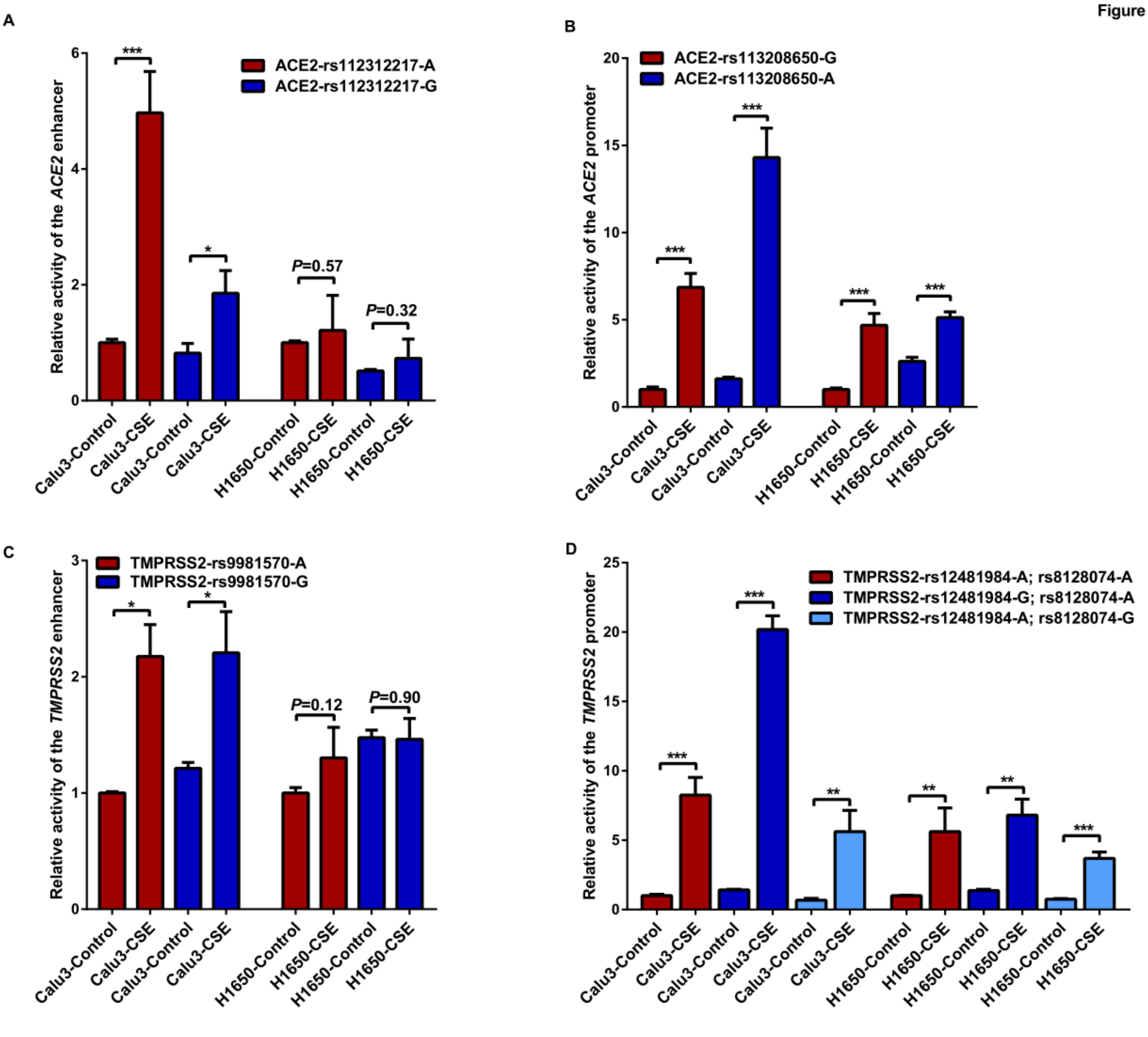

Figure S2. Effects of single nucleotide polymorphisms (SNPs) on the activities of the ACE2 and TMPRSS2 transcriptional regulatory sequences in cells stimulated with CSE

(A) The activity of the *ACE2* enhancer with A (served as reference) or G at rs112312217. (B) The activity of the *ACE2* promoter with A (served as reference) or G at rs113208650. (C) The activity of the *TMPRSS2* enhancer with A (served as reference) or G at rs9981570. (D) The activity of the *TMPRSS2* promoter with A (served as reference) and G at rs12481984 and rs812074. Control, cells stimulated with DMSO (0.1%, 12 hours). CSE, cells stimulated with 20% CSE for 12 hours. The luciferase activity of promoter/enhancer sequence with reference genotype, which was detected in cells treated with DMSO, served as a reference to calculated relative luciferase activity. Student's *t*-test, $^{*}P<0.05$, $^{**}P<0.005$, $^{***}P<0.0005$. Error bars represent standard deviation.

Figure S3

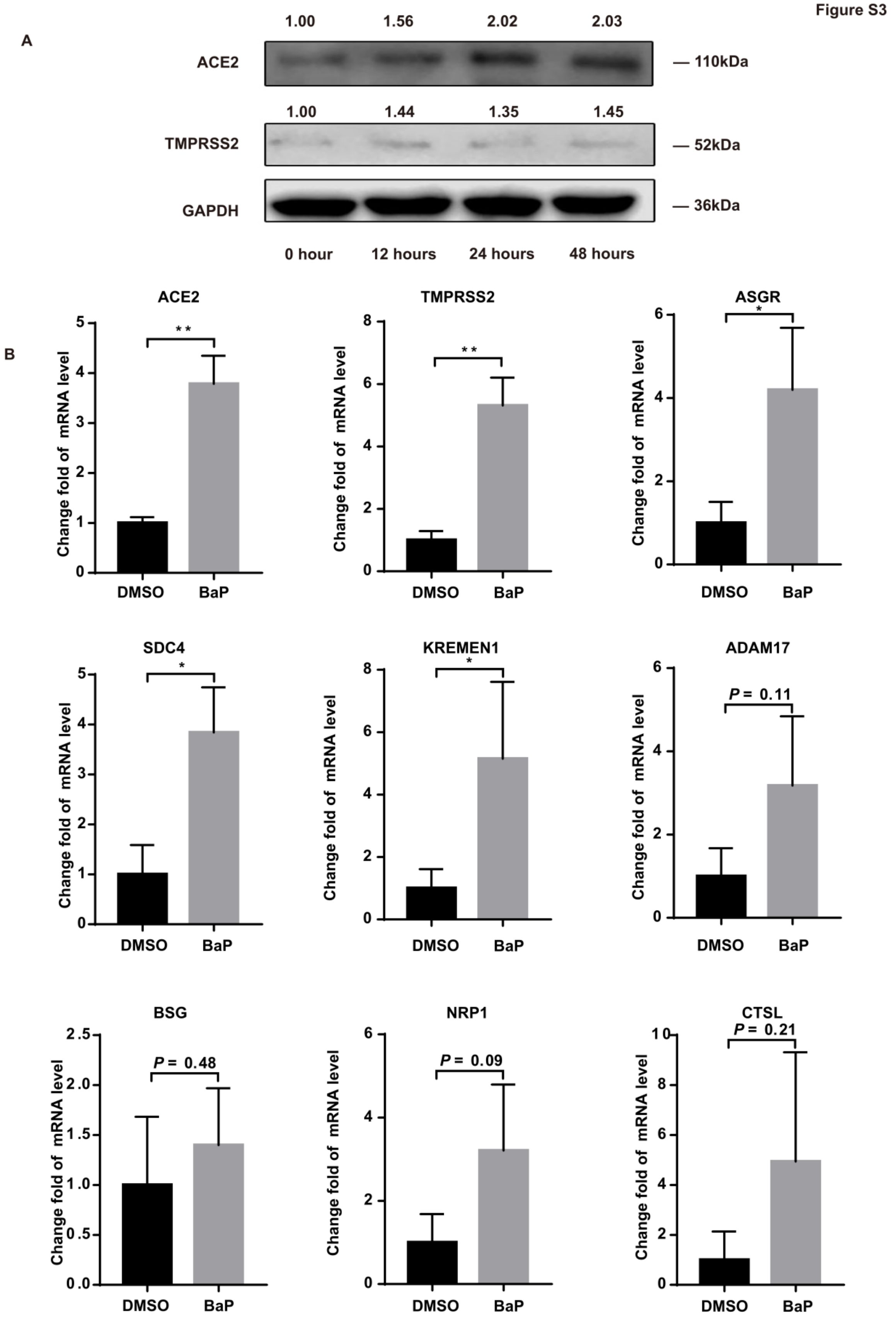
A
1.00
1.56
2.02
2.03
ACE2
110kDa
1.00
1.44
1.35
1.45
TMPRSS2
52kDa
GAPDH
36kDa
0 hour
12 hours
24 hours
48 hours
B
ACE2
TMPRSS2
ASGR
SDC4
KREMEN1
ADAM17
BSG
NRP1
CTSL
Change fold of mRNA level
DMSO
BaP
P = 0.11
P = 0.48
P = 0.09
P = 0.21

Figure S3. BaP upregulated the expression of SARS-CoV-2 receptors in human primary human alveolar type Ⅱ (AT Ⅱ) cells

(A) Representative image of Western blot tests detecting the protein levels of ACE2 and TMPRSS2 in primary AT Ⅱ cells. Cells were stimulated with BaP at 15μM for 0, 12, 24, and 48 hours before being subjected to Western blot tests. (B) Representative results of RT-qPCR evaluating SARS-CoV-2 receptors in primary AT Ⅱ cells. Cells were stimulated with BaP at 15μM or 0.1% DMSO for 12 hours. All experiments were repeated three times with three replicates and one of the representative results is shown here. RT-qPCR data is normalized by reference gene GAPDH. The intensity of Western blot band is measured by Image J software and normalized to the band intensity of GAPDH. Relative mRNA level and protein level are presented as a ratio of BaP group to DMSO group. Student's *t*-test, *P<0.05, **P<0.005, ***P<0.0005. Error bars represent standard deviation.

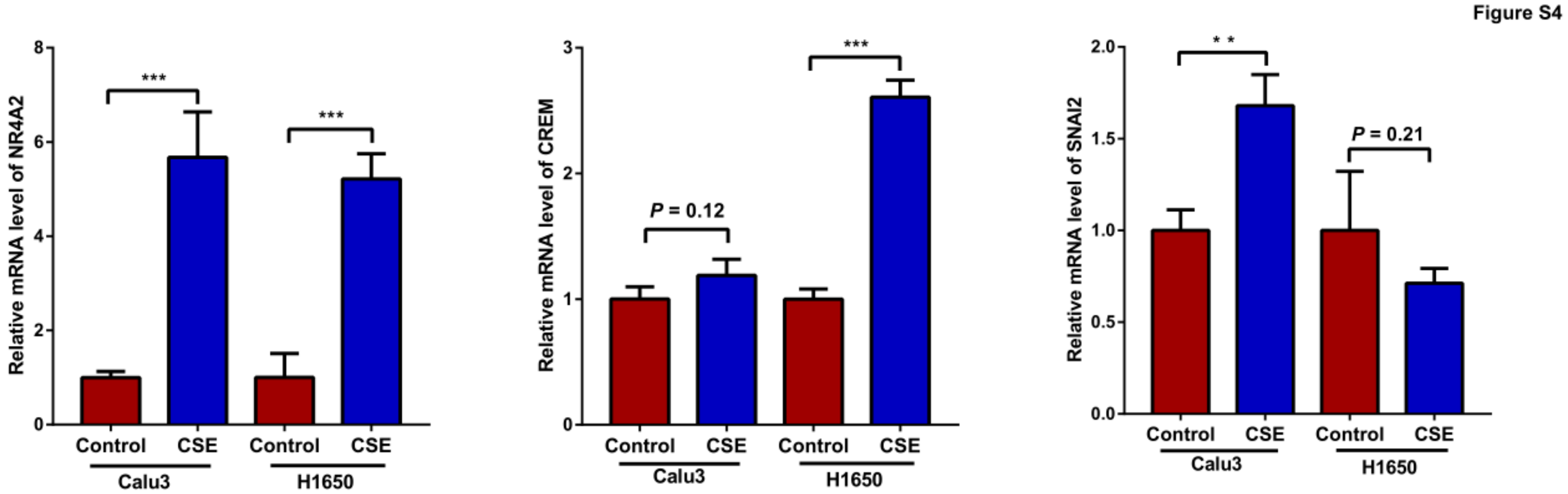

Figure S4. Effects of cigarette smoke extract (CSE) on the transcriptional levels of NR4A2, CREAM, and SNAI2 in Calu3 and H1650 cell lines

Results of RT-qPCR tests evaluating the transcriptional levels of *NR4A2*, *CREAM*, and *SNAI2* in cells stimulated with 20% CSE for 12 hours. All experiments were repeated three times with three replicates and one of the representative results is shown here. RT-qPCR data is normalized by reference gene GAPDH. Relative mRNA level is presented as a ratio of the CSE group to the control group. Student's *t*-test, $^{*}P<0.05$, $^{**}P<0.005$, $^{***}P<0.0005$. Error bars represent standard deviation.

Figure S5

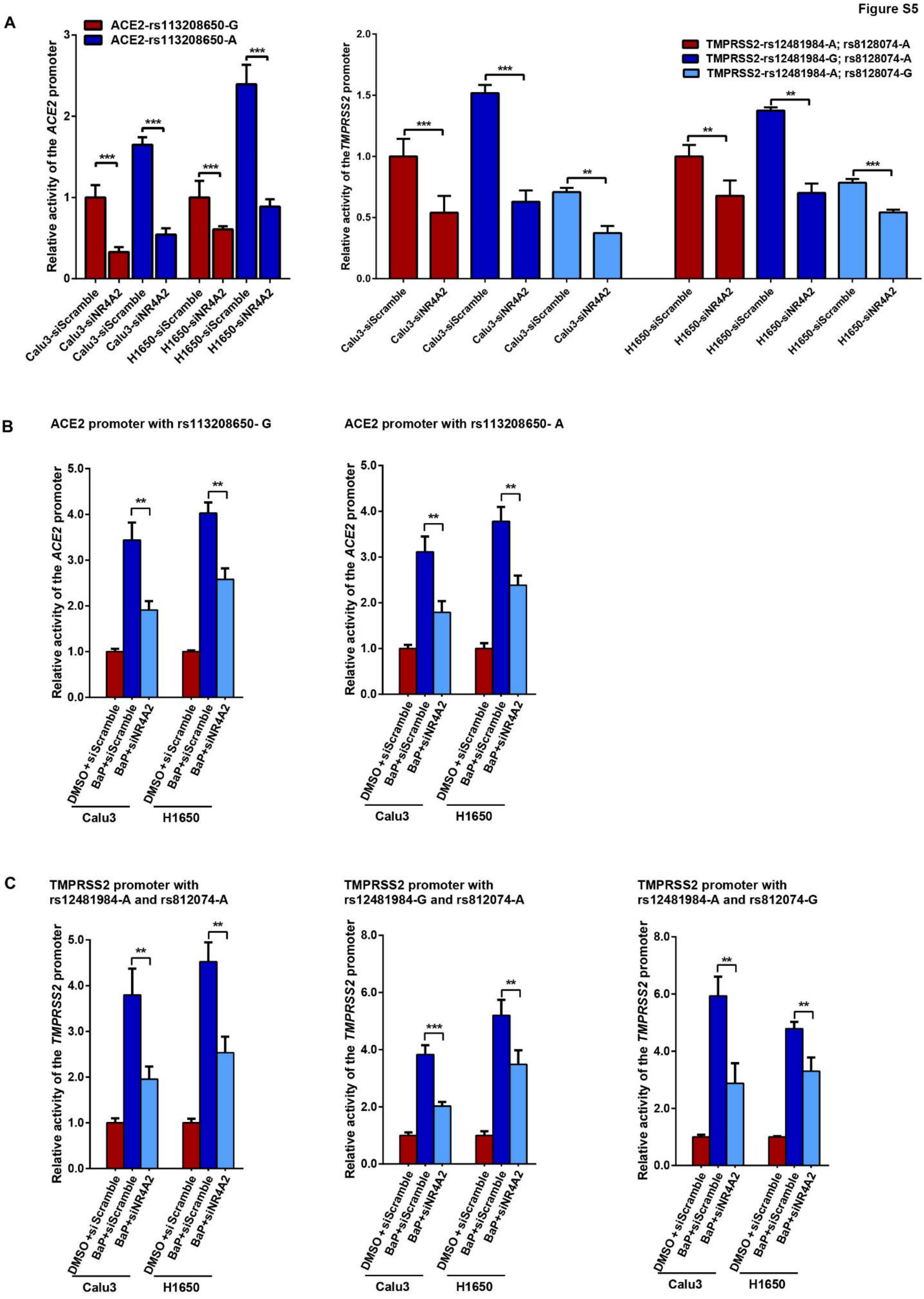
A
ACE2-rs113208650-G
ACE2-rs113208650-A
Relative activity of the ACE2 promoter
Calu3-siScramble
Calu3-siNR4A2
H1650-siScramble
H1650-siNR4A2
TMPRSS2-rs12481984-A; rs8128074-A
TMPRSS2-rs12481984-G; rs8128074-A
TMPRSS2-rs12481984-A; rs8128074-G
Relative activity of the TMPRSS2 promoter
B
ACE2 promoter with rs113208650- G
ACE2 promoter with rs113208650- A
DMSO+siScramble
BaP+siScramble
BaP+siNR4A2
Calu3
H1650
C
TMPRSS2 promoter with rs12481984-A and rs812074-A
TMPRSS2 promoter with rs12481984-G and rs812074-A
TMPRSS2 promoter with rs12481984-A and rs812074-G

Figure S5. Effects of SNPs on the activities of the *ACE2* and *TMPRSS2* transcriptional regulatory sequences in cells with NR4A2 knockdown

(A) NR4A2 knockdown significantly decreased the activity of the *ACE2* promoter sequences with different genotypes at rs113208650. NR4A2 knockdown significantly decreased the activities of the *TMPRSS2* promoter sequences with different genotypes at rs12481984 and rs8128074. (B) NR4A2 knockdown attenuated the positive effect of BaP on the activity of the *ACE2* promoter sequences with different genotypes at rs113208650. (C) NR4A2 knockdown attenuated the positive effect of BaP on the activity of the *TMPRSS2* promoter sequences with different genotypes at rs12481984 and rs8128074. siScramble, cells transfected with siRNA targeting scramble sequence for 48 hours. siNR4A2, cells transfected with siRNA targeting *NR4A2* for 48 hours. Luciferase activity of cells treated with DMSO and transfected with siScramble was applied as a reference to calculate relative luciferase activity. Student's *t*-test, $^{*}P<0.05$, $^{**}P<0.005$, $^{***}P<0.0005$. Error bars represent standard deviation.

Figure S6

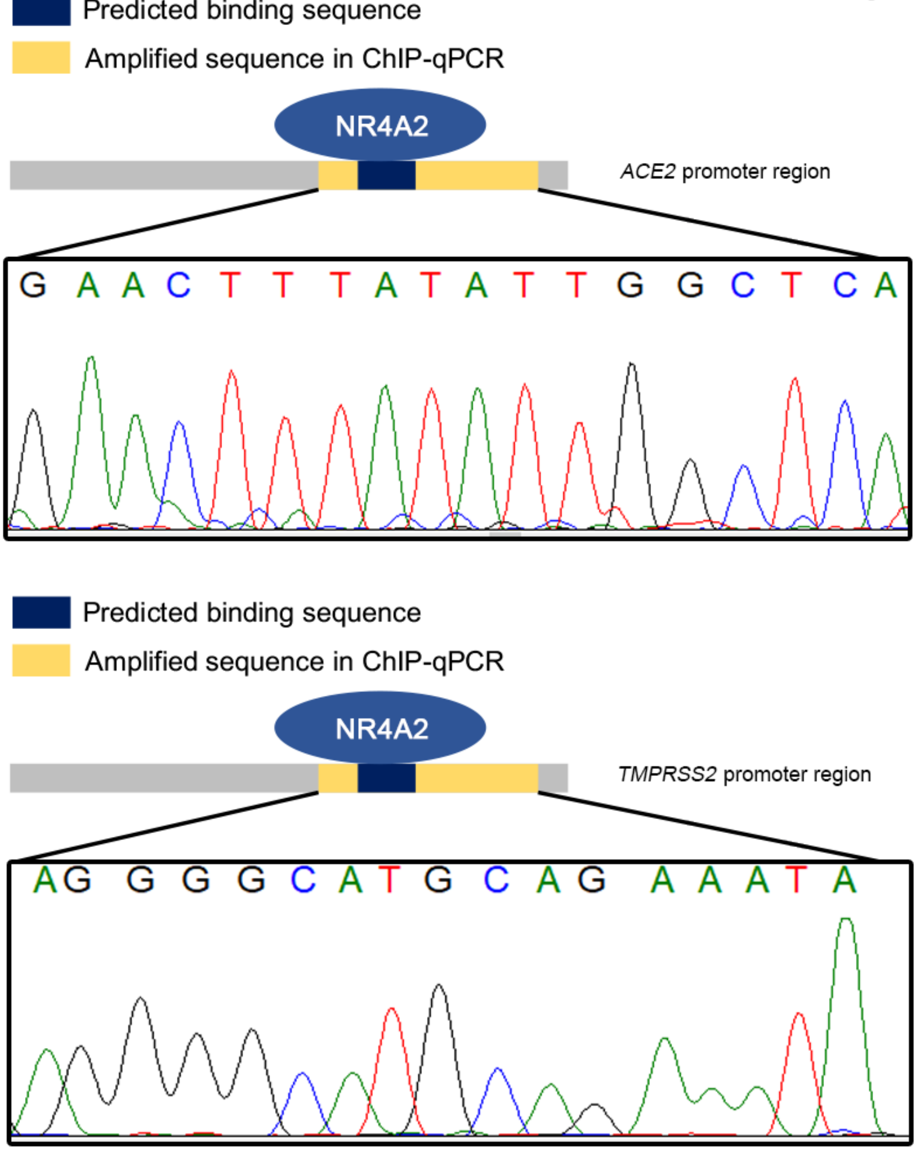

Figure S6. ChIp-qPCR followed Sanger sequencing

The representative image of Sanger sequencing chromatogram (the result of Calu3 cells). The product of qPCR conducted with DNA binding to NR4A2 was subjected to Sanger sequencing. The sequence of amplified ChIP-qPCR products was confirmed to be the ACE2 and TMPRSS2 promoter region.

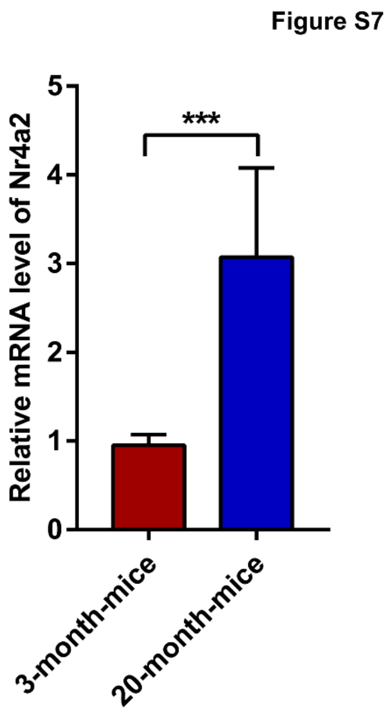

Figure S7. mRNA level of *Nr4a2* in lung tissues from mice

The lung tissues from 3-month old mice and 20-month old mice (n = 3 per group) were subjected to RT-qPCR. Relative mRNA level was calculated as a ratio of the value of 20-month old mice to 3-month old mice. Student's *t*-test, $^{*}P<0.05$, $^{**}P<0.005$, $^{***}P<0.0005$. Error bars represent standard deviation.

Figure S8

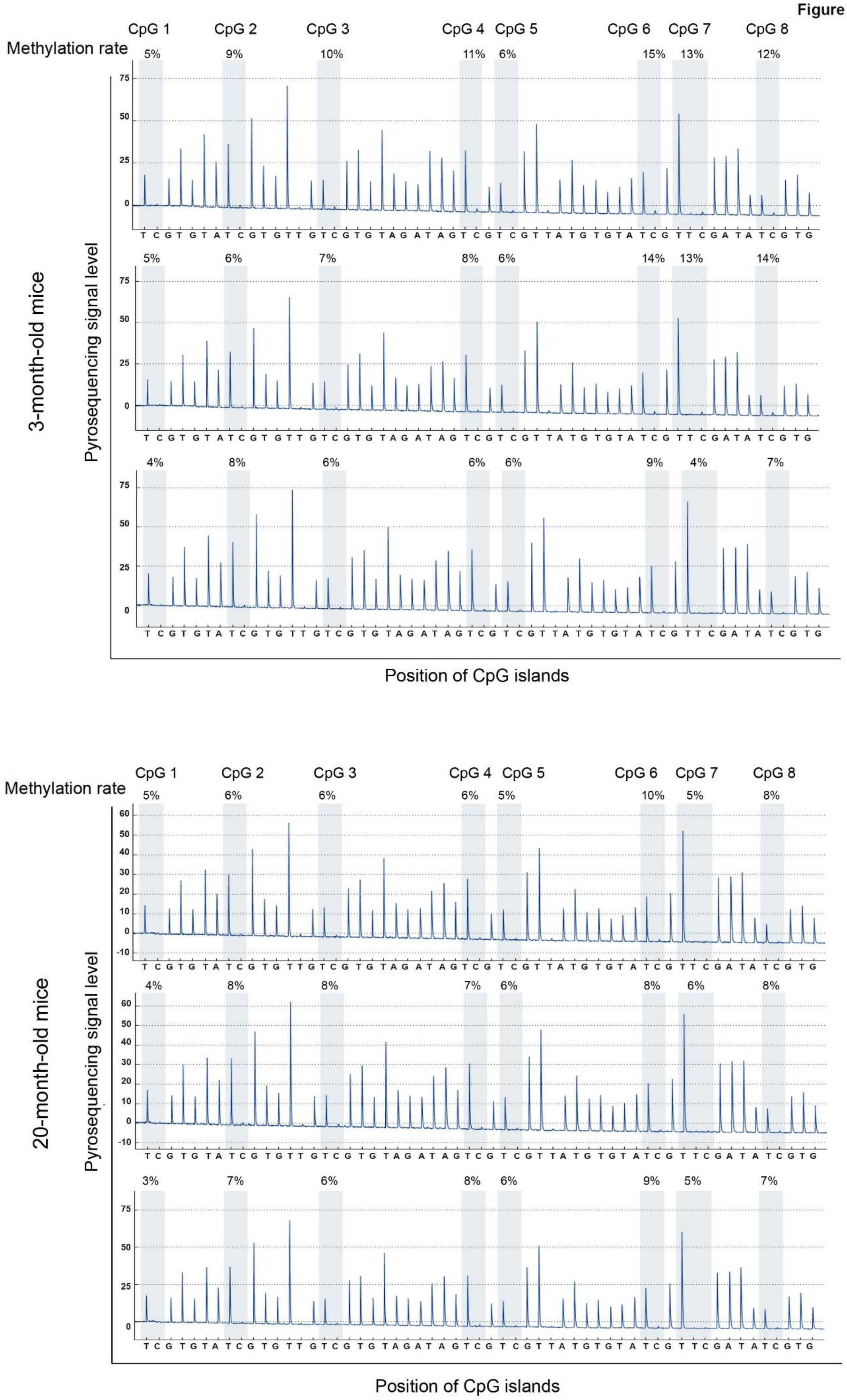
CpG 1
CpG 2
CpG 3
CpG 4
CpG 5
CpG 6
CpG 7
CpG 8
Methylation rate
5% 9% 10% 11% 6% 15% 13% 12%
5% 6% 7% 8% 6% 14% 13% 14%
4% 8% 6% 6% 6% 9% 4% 7%
3-month-old mice
Pyrosequencing signal level
Position of CpG islands
5% 6% 6% 6% 5% 10% 5% 8%
4% 8% 8% 7% 6% 8% 6% 8%
3% 7% 6% 8% 6% 9% 5% 7%
20-month-old mice
Pyrosequencing signal level
Position of CpG islands

Figure S8. The pyrosequencing data of mouse lung tissues at the *Nr4a2* promoter region

Genomic DNA extracted from lung tissues of 3-month-old mice and 20-month-old mice were subjected to the pyrosequencing assay. The pyrosequencing signal of each nucleotide site was shown on the X axis. The sequence of the *Nr4a2* promoter was shown on the Y axis. The light blue region represented the position of CpG islands. The methylation rate was marked on the top of the CpG island region.

Figure S9

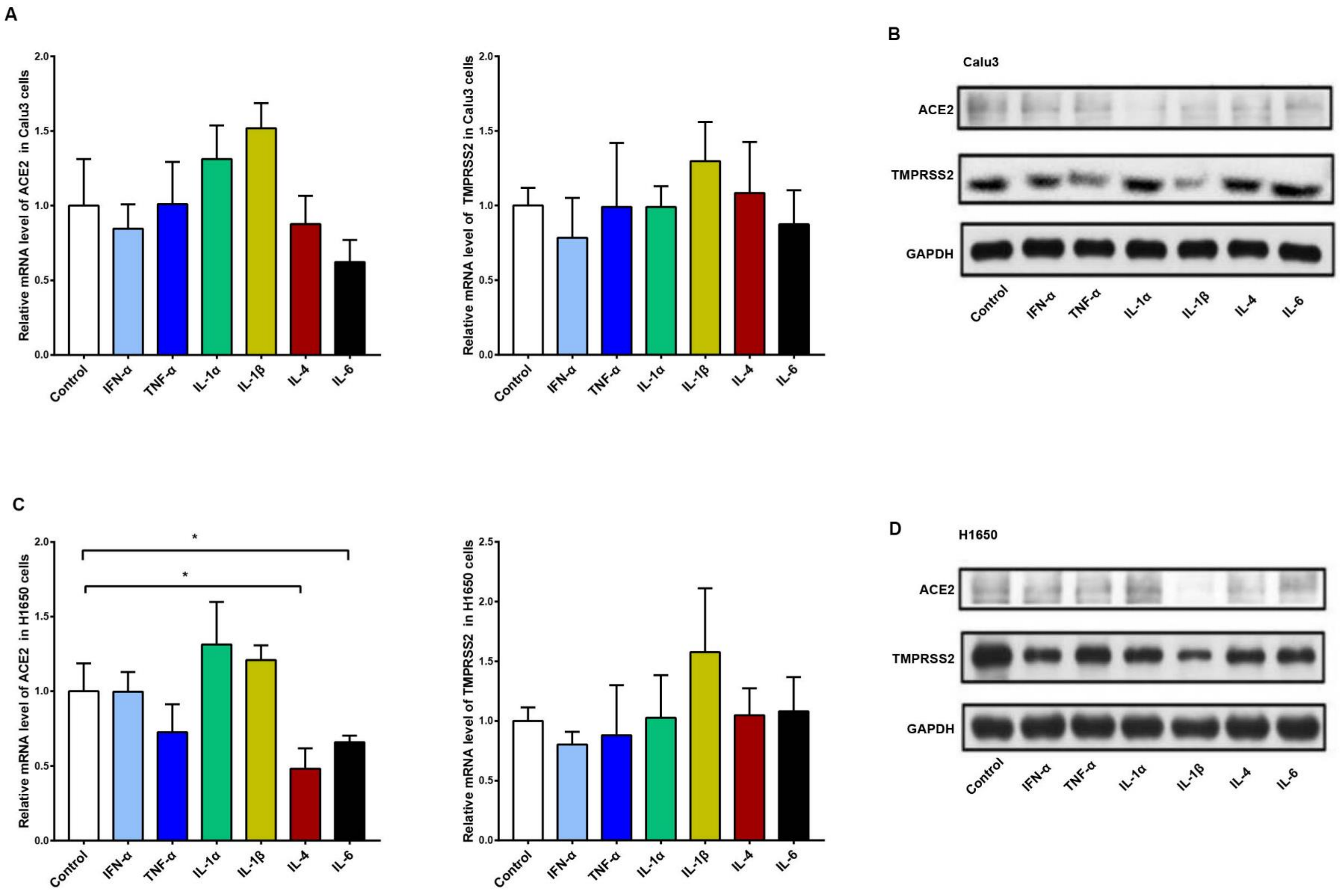
A
Relative mRNA level of ACE2 in Calu3 cells
Relative mRNA level of TMPRSS2 in Calu3 cells
2.0
1.5
1.0
0.5
0.0
Control
IFN-α
TNF-α
IL-1α
IL-1β
IL-4
IL-6
B
Calu3
ACE2
TMPRSS2
GAPDH
C
Relative mRNA level of ACE2 in H1650 cells
Relative mRNA level of TMPRSS2 in H1650 cells
2.5
*
D
H1650

Figure S9. Effects of inflammatory factors on the expression of ACE2 and TMPRSS2

(A) The mRNA levels of ACE2 and TMPRSS2 in Calu3 cells treated with inflammatory factors (50ng/ml, 12 hours), respectively. (B) The protein levels of ACE2 and TMPRSS2 in Calu3 cells treated with inflammatory factors (50ng/ml, 12 hours), respectively. (C) The mRNA levels of ACE2 and TMPRSS2 in H1650 cells treated with inflammatory factors (50ng/ml, 12 hours), respectively. (D) The protein levels of ACE2 and TMPRSS2 in H1650 cells treated with inflammatory factors (50ng/ml, 12 hours), respectively. All experiments were repeated three times with three replicates and one of the representative results is shown here. RT-qPCR data is normalized by reference gene GAPDH and relative mRNA level is presented as a ratio of inflammatory factors treated group to control group. The intensity of Western blot band is measured by Image J software and normalized to band intensity of GAPDH. Relative band intensity is presented as a ratio of inflammatory factors treated group to control group. Student's t-test, $^{*}P<0.05$, $^{**}P<0.005$, $^{***}P<0.0005$. Error bars represent standard deviation.

Figure S10

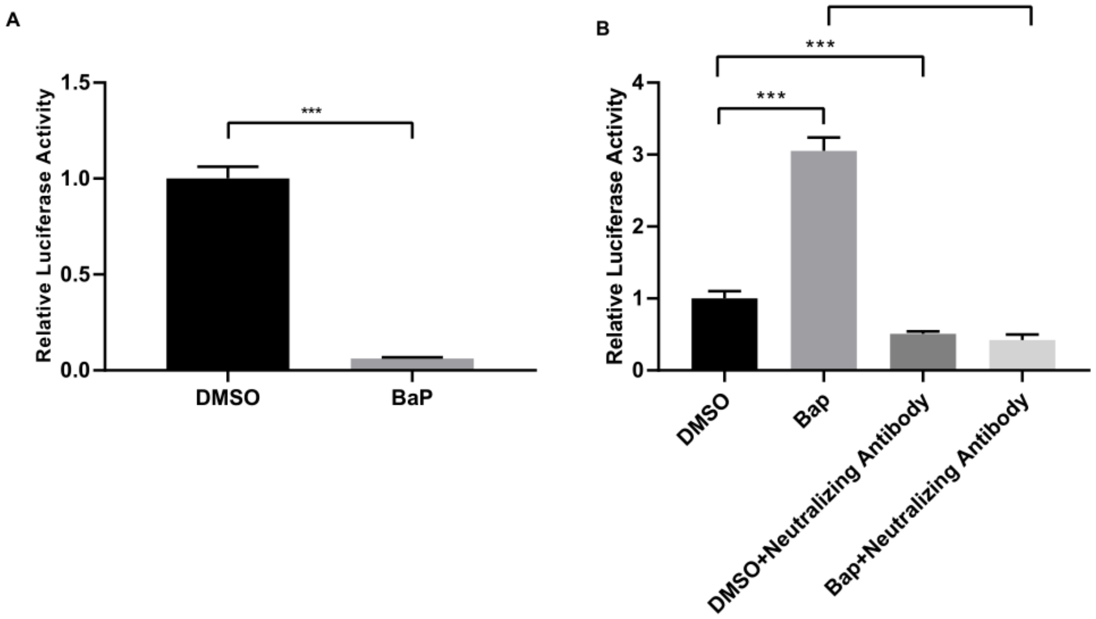

Figure S10. Infection assay of VSV-based pseudoviruses of SARS-CoV-2

Viral infection was evaluated by detecting luciferase activity. (A) Effect of BaP on infection of VSV-based pseudovirus carrying G protein (control pseudoviruses of pseudoviruses for SARS-CoV-2). Calu3 cells were treated with 15μM BaP or 0.1% DMSO for 12 hours. Then, cells were infected with control pseudoviruses for 24 hours before being subjected to luciferase assay. (B) Effect of anti-Spike antibody on infection of VSV-based pseudovirus of SARS-CoV-2. Calu3 cells were categorized into four groups and treated with 0.1% DMSO, 15μM BaP, 0.1% DMSO combined with anti-Spike antibody, and 15μM BaP combined with anti-Spike antibody, respectively. Twelve hours later, cells were infected with VSV-based pseudoviruses of Omicron BA.5 for 24 hours and then subjected to luciferase assay. Student's *t*-test, $^{*}P<0.05$, $^{**}P<0.005$, $^{***}P<0.0005$. Error bars represent standard deviation.

Figure S11

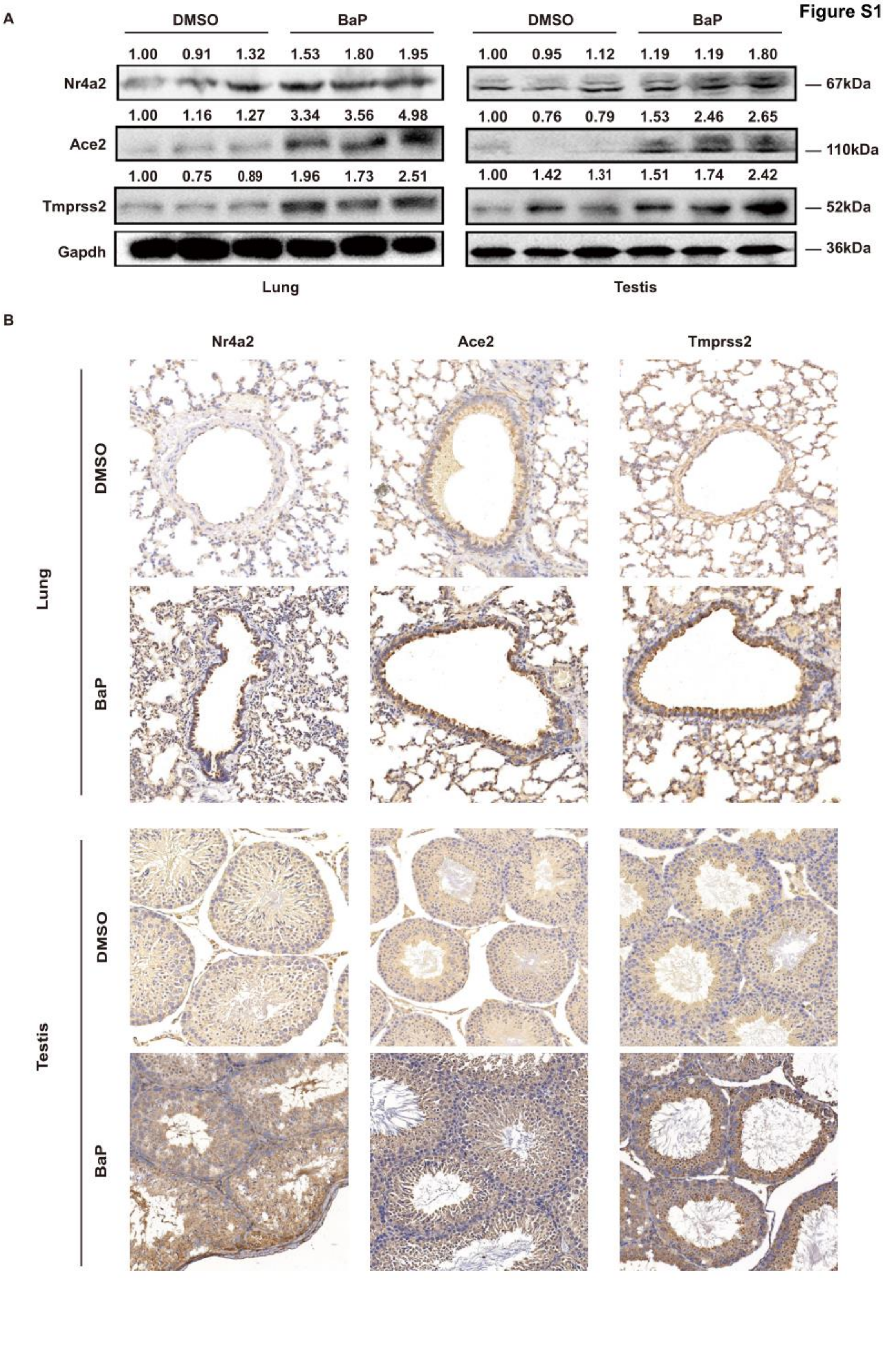
A
DMSO
BaP
DMSO
BaP
1.00 0.91 1.32 1.53 1.80 1.95
1.00 0.95 1.12 1.19 1.19 1.80
Nr4a2
— 67kDa
1.00 1.16 1.27 3.34 3.56 4.98
1.00 0.76 0.79 1.53 2.46 2.65
Ace2
— 110kDa
1.00 0.75 0.89 1.96 1.73 2.51
1.00 1.42 1.31 1.51 1.74 2.42
Tmprss2
— 52kDa
Gapdh
— 36kDa
Lung
Testis
B
Nr4a2
Ace2
Tmprss2
Lung
DMSO
BaP
Testis
DMSO
BaP

Figure S11. Effective of BaP on the expression of Nr4a2, Ace2, and Tmprss2 in lung and testis tissues of hamsters

(A) Representative image of Western blot tests. (B) Representative image of IHC (scale bar, 50μm). Hamsters in BaP group (n = 3) were injected with BaP (125mg/kg) into the left pleural cavity and scrotum. The hamsters in DMSO group were injected with the equal value of DMSO in the same way. Three weeks later, lung and testis tissues were collected and subjected to Western blot and IHC analysis. The intensity of Western blot band is measured by Image J software and normalized to band intensity of GAPDH. Relative band intensity is presented as a ratio of BaP group to DMSO group.

Figure S12

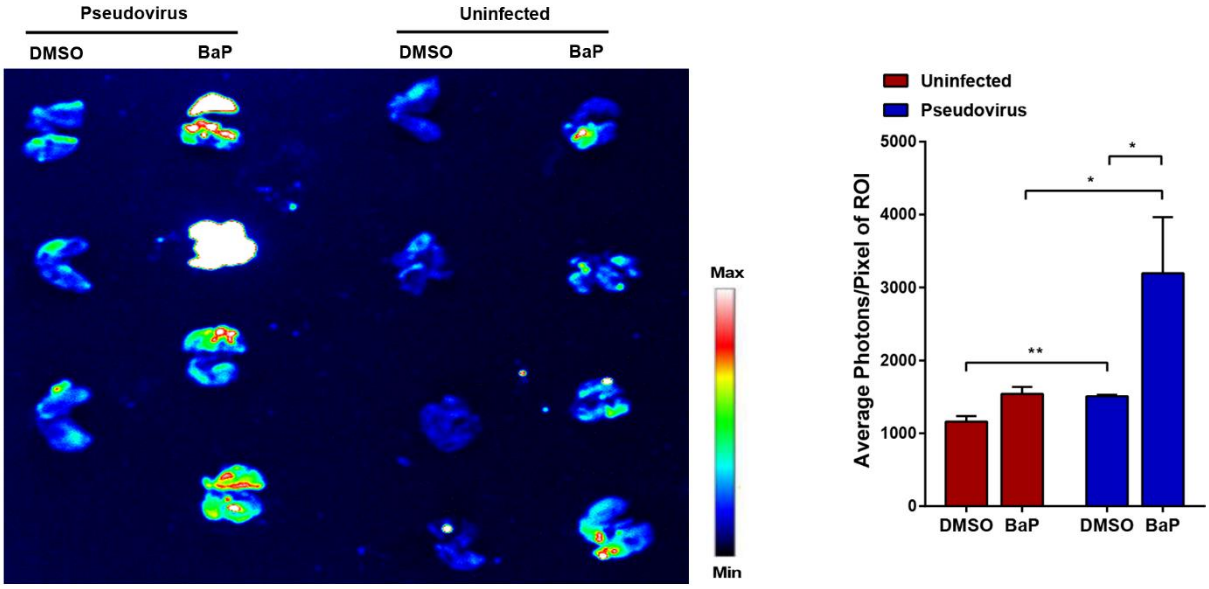

Figure S12. BaP facilitated the infection of SARS-CoV-2 pseudovirus in lung tissues of mice

Ex vivo live images and quantitative analysis of image data for lung tissues of healthy C57BL/6 mice (n = 3 per group). C57BL/6 mice were injected with BaP (125mg/kg) or DMSO into left pleural cavity. Three weeks later, mice were injected with lentivirus-based Omicron pseudovirus into left pleural cavity (100 μL, 2×10^7U/mL). GFP fluorescence was detected by two days later. The flux of GFP fluorescence in the region of interest (ROI) was measured to evaluate the infection level of pseudovirus. Student's *t*-test, *P<0.05, **P<0.005, ***P<0.0005. Error bars represent standard deviation.

Supplementary Table S1. Predictive results of transcription factors binding to promoters of *ACE2* and *TMPRSS2**

ACE2			TMPSS2		
Transcription factor	Relative score or Peak value of ChIP-seq	Predicted binding site (sequence or location)	Transcription factor	Relative score or Peak value of ChIP-seq	Predicted binding site (sequence or location)
Predictive results derived from JASPAR*			SNAI2	1	GGCAGGTGC
NR4A2	1.00	AAGGTCAC	ID4	0.99	TGCACCTGCC
SNAI2	0.98	TACAGGTGC	NRL	0.97	GATCTGCTGAC
ID4	0.97	GGCACCTGTA	OTX2	0.96	TTAATCTC
NRL	0.95	AATCTGCTGAG	TCF3	0.99	CACACCTGTA
OTX2	1.00	TTAATCCT	TCF4	0.99	CACACCTGTA
TCF3	0.97	GGCACCTGTA	GATA3	0.98	AGATAAAA
TCF4	0.97	GGCACCTGTA	HIC2	0.95	ATGCCAGCC
GATA3	0.98	AGATAAAA	NFIX	1	GGTGCCAAA
HIC2	1.00	GTGCCCACC	NFIA	0.98	GGTGCCAAAG
STAT1	0.99	TTTCTAGGAAA	TFAP2A	0.97	AGCCCTCGGGCA
TCF7L2	0.97	ACAGATCAAAGAGA	KLF5	0.98	TCCCCGCCCC
STAT3	0.99	TTTCTGGGAAT	TFAP2C	0.97	AGCCCTCGGGCA
USF1	0.96	TCCACGTGACC	ZEB1	0.99	ACTCACCTG
FOXO3	1.00	GTAAACAA	TFAP2B	0.97	AGCCCTCGGGCA
FOXG1	1.00	GTAAACAA	SP1	0.98	CCCCCGCCCCC
FOXP2	0.95	CAGTAAACAAT	EGR1	1	CCCCCGCCCCCGCC

FOXO6	1.00	GTAAACA	FIGLA	0.95	CACACCTGTA
FOXI1	1.00	GTAAACA	NKX2-3	0.96	GGCACTTGAG
TEAD3	1.00	ACATTCCA	NKX2-8	0.99	GCACTTGAG
FOXO4	1.00	GTAAACA	TBX5	1	AGGTGTGA
TEAD4	0.99	CACATTCCAG	E2F6	0.95	GGGCGGGAGTG
FOXD1	0.99	GTAAACAA	SP3	0.96	CCCCCGCCCCC
FOXL1	1.00	GTAAACA	TBX4	1	AGGTGTGA
TEAD1	0.97	CACATTCCAG	RUNX3	0.96	AGACCACAAA
MNT	0.96	TCCACGTGAC	E2F4	0.96	CGGCGGGAGGA
HMBOX1	0.96	GTTAGTTAAG	MGA	1	AGGTGTGA
SRY	1.00	GTAAACAAT	KLF16	0.95	CCCCCGCCCCC
FOXD2	1.00	GTAAACA	PHOX2A	0.95	TAATTTAATTT
NFATC3	0.95	ATTTTCCGTG	TBX1	1	AGGTGTGA
FOXP3	1.00	GTAAACA	TBX15	1	AGGTGTGA
CDX1	0.95	GCCATAAAG	SP2	0.96	CCCCCGCCCCCGCCC
MEIS3	0.98	GTGACAGG			
MEIS2	0.95	GTGACAGG			
GATA5	0.99	TGATAAGA			
NKX6-1	0.96	TTCATTAA			
MEIS1	1.00	ATGACAG			

RHOXF1	1.00	ATGATCCC			
DLX6	0.97	ACAATTAT			
Predictive results derived from RegulomeDB[†]					
MYBL2	43.19	chrx: 15620005-15620621	ZNF770	48.03	chr21: 42881181-42881561
USF1	31.59	chrx: 15620023-15620223	EZH2	805.03	chr21: 42877162-42882158
CREM	31.06	chrx: 15620060-15620584	CTCF	523.43	chr21: 42880802-42881613
SAP130	50.92	chrx: 15620043-15620579	ZNF121	137.70	chr21: 42881086-42881522
ZNF614	40.59	chrx: 15620046-15620536	AR	23.00	chr21: 42880733-42881320
MNT	153.03	chrx: 15620013-15620222	MAFK	23.50	chr21: 42880843-42881083
FOXA2	90.80	chrx: 15620016-15620416	TAF1	12.53	chr21: 42880599-42881089
NCOR1	32.21	chrx: 15620045-15620615			
ARID3A	23.21	chrx: 15619992-15620368			
KAT8	30.86	chrx: 15620044-15620608			

*****Transcription factors were predicted by using JASPAR database. The relative score and putative binding sequences were listed here. The robust results were identified as those with relative score >0.95.

[†] Transcription factors were predicted by using RegulomeDB ChIP-seq database. The peak value of ChIP-seq and location were listed here.

Supplementary Table S2. The effects of cigarette exposure on the expression of transcription factors [median (interquartile ranges)]

Genes	GSE994			GSE10718		GSE8987		LUAD		
	Never Smoker	Former Smoker	Current Smoker	Cells exposed to air	Cells exposed to smoke	Never Smoker	Current Smoker	Never Smoker	Smoke ≤15years	Smoke >15years
CREM	69.10 (57.45,98.05)	75.00 (61.05,101.33)	82.45 (73.50,89.35)	**568.19[3] (514.35,633.25)**	**1349.01[3] (1075.74,1626.01)**	278.75 (238,396.61)	640.48 (496.79,727.16)	**2.02[4] (1.82,2.29)**	**2.29[4] (1.97,2.63)**	2.13 (1.89,2.46)
NR4A2	68.10 (53.85,106.75)	82.25 (49.45,101.43)	73.80 (52.63,95.85)	**33.08[3] (23.62,60.2)**	**140.07[3] (99.59,170.25)**	378.46 (259.75,431.82)	321.68 (223.91,334.68)	**2.31[4] (1.6,3.12)**	**2.79[4] (1.93,3.79)**	2.67 (1.85,3.81)
SNAI2	**44.50[1] (37.05,53.2)**	**25.00[1,2] (8.175,44.93)**	**40.90[2] (29.25,58.43)**	13060.60 (12117,13659.73)	12745.7 (9831.6,13490.93)	455.4 (239.13,507.04)	382.3 (327.74,535.21)	2.02 (1.48,2.76)	**2.2[5] (1.66,2.8)**	**1.83[5] (1.37,2.47)**

[1] Former Smoker vs. Never Smoker, *P*<0.05
[2] Current Smoker vs. Former Smoker, *P*<0.05.
[3] Cells exposed to smoke vs. Cells exposed to air, *P*<0.05.
[4]. Smoke ≤15 years vs. Never smoker, *P*<0.05.
[5] Smoke >15 years vs. Smoke ≤15 years, *P*<0.05.

Supplementary Table S3. Peak values of transcriptional regulatory region of NR4A2, ACE2, and TMPRSS2

Gene	Transcription regulatory region detected by ATAC-seq	Distance to TSS	Average of peak value		*P* value
			DMSO	BaP	
NR4A2	Chr2: 156331898-156333541	-821bp to +822bp	645.36	755.89	0.0067
	Chr2: 156334042-156335108	-2836 bp to -1320 bp	275.57	359.02	0.0096
ACE2	Chr21 41506833-41509172	-1107 bp to +1232bp	332.05	355.13	0.41
TMPRSS2	ChrX 15579863-15580587	-167bp to +557bp	58.01	51.720	0.46

TSS: transcription start site; DMSO, BaP: cells treated with 0.4% DMSO or 15μM BaP for 12-h (n=3 per group)

Supplementary Table S4. Association between age and methylation level of CpG islands within translational regulation regions of NR4A2

CpG island	Location	Function region	Distance to TSS (bp)	β with Age	*P* value
cg25247969	chr2:(157188744-157188746)	5'UTR	-487	-0.0505	0.67
**cg09408520**	**chr2:(157188964-157188966)**	**5'UTR**	**-267**	**-0.2850**	**0.01**
cg15699971	chr2:(157189240-157189242)	promoter	9	-0.1523	0.20
cg20804199	chr2:(157189307-157189309)	promoter	76	-0.0051	0.97
cg18881247	chr2:(157189313-157189315)	promoter	82	-0.1363	0.25
cg16246410	chr2:(157189315-157189317)	promoter	84	-0.0550	0.64
cg20945253	chr2:(157189509-157189511)	promoter	278	-0.0972	0.41
cg03953709	chr2:(157189667-157189669)	promoter	436	-0.0954	0.42
cg01123282	chr2:(157189754-157189756)	promoter	523	-0.0688	0.56
cg07646377	chr2:(157189968-157189970)	promoter	737	-0.1582	0.18
cg23474904	chr2:(157190107-157190109)	promoter	876	-0.1390	0.24
cg13945301	chr2:(157190181-157190183)	promoter	950	-0.0842	0.48
cg11379337	chr2:(157190310-157190312)	promoter	1079	-0.0785	0.51
**cg16151636**	**chr2:(157190746-157190748)**	**promoter**	**1515**	**-0.2480**	**0.03**
cg05694870	chr2:(157190957-157190959)	promoter	1726	-0.1553	0.19
cg21086083	chr2:(157191260-157191262)	promoter	2029	-0.1472	0.21
cg26872149	chr2:(157191395-157191397)	promoter	2164	-0.1731	0.14
cg12335829	chr2:(157192127-157192129)	promoter	2896	-0.0155	0.90
cg11554249	chr2:(157195306-157195308)	promoter	6075	0.0591	0.62
cg22391814	chr2:(157195516-157195518)	promoter	6285	0.0603	0.61
**cg00484803**	**chr2:(157196298-157196300)**	**promoter**	**7067**	**-0.2310**	**0.04**
cg21512275	chr2:(157198068-157198070)	promoter	8837	-0.1003	0.40
cg18822446	chr2:(157198282-157198284)	promoter	9051	-0.1137	0.33
cg15053019	chr2:(157198335-157198337)	promoter	9104	-0.1445	0.22
cg21008684	chr2:(157198369-157198371)	promoter	9138	-0.1923	0.10
**cg09358454**	**chr2:(157198725-157198727)**	**promoter**	**9494**	**-0.2790**	**0.02**
cg24023498	chr2:(157199344-157199346)	promoter	10113	-0.0348	0.77
cg19878911	chr2:(157199399-157199401)	promoter	10168	-0.2187	0.06
cg15369144	chr2:(157199485-157199487)	promoter	10254	-0.1314	0.26

Supplementary Table S5. Primers for RT-qPCR

Gene	Forward primer	Reverse primer
ACE2	5’-CGAAGCCGAAGACCTGTTCTA-3	5’-GGGCAAGTGTGGACTGTTCC-3’
TMPRSS2	5’- GTCCCCACT GTCTACGAGGT -3	5’- CAGACGACGGGGTTGGAAG -3’
NR4A2	5’- GTTCAGGCGCAGTATGGGTC -3’	5’- CTCCCGAAGAGTGGTAACTGT -3’
NR4A2（MICE）	5’- AACCCTGACTATCAAATGAGTG -3’	5’- CAATGCAGGAGAAGGCAGAAAT -3’
CREM	5’-GGGCAAGTGTGGACTGTTCC-3’	5’-GGGCAAGTGTGGACTGTTCC-3’
SNAI2	5’- ATGATCGGCTTAATCTGCCTG -3’	5’- TCCGGGTTGTTCTTTCTGTCC -3’
GAPDH	5’- CTGGACCGTCTCAAGGTGTT -3’	5’- GCCCCAGATAGGCAAACTT -3’
GAPDH（MICE）	5’- AGGTCGGTGTGAACGGATTTG -3’	5’- TGTAGACCATGTAGTTGAGGTCA -3’
NRP1	5’-CCCAACAGCCTTGAATGCAC-3’	5’-ATTTCTAGCCGGTCGTAGCG-3’
ASGR1	5’-GAGAGAGACGTTCAGCAACTTC-3’	5’-GGGACTCTAGCGACTTCATCTT-3’
SDC4	5’-GGCAGGAATCTGATGACTTTG -3’	5’-GGCCGATCATGGAGTCTTC-3’
CTSL	5’-ATGAATCCTACACTCATCCTTGCTGCCTTT -3’	5’-CCACACTGCTCTCCTCCATCCTTCTT-3’
BSG	5’-AGGGAGATTTGGTATTTTATTTTTT-3’	5’-ACCTAACTATCTCTCCACATCCTAC-3’
KREMEM1	5’-CAGCCCCGATGCATCCT -3’	5’-TCCGCGTATAAGTGCTTGTGA-3’
ADAM17	5’-CGGATGGTCTAGCAGAATG -3’	5’-GGGCCTTACTTTCAATGGT -3’

Supplementary Table S6. Antibodies for Western blot, IHC, and ChIP analysis

Proteins	Antibodies	Source	Catalog number	Dilution ratio	References
ACE2 (for western blot and IHC)	Anti- ACE2 antibody	SAB	#5347	WB：1:1000<br>IHC：1:500	This antibody has not yet been referenced specifically in any publications.
TMPRSS2 (for western blot and IHC)	Anti- TMPRSS2 antibody	SAB	#54629	WB：1:1000<br>IHC：1:500	This antibody has not yet been referenced specifically in any publications.
NR4A2 (for western blot, IHC and ChIP)	Anti- NR4A2 antibody	Proteintech	10975-2-ap	WB：1:1000<br>IHC：1:500	Chen X, et al. Caveolin-1 facilitates cell migration by upregulating nuclear receptor 4A2/retinoid X receptor α-mediated β-galactoside α2,6-sialyltransferase I expression in human hepatocarcinoma cells. Int J Biochem Cell Biol. 2021 ;137:106027.
SFTPB (for IHC)	SFTPB Conjugated antibody	SAB	#C30048	1:200	This antibody has not yet been referenced specifically in any publications.
GAPDH	Anti-GAPDH antibody	Abcam	ab181602	1:2000	Zhou HL, et al. Metabolic reprogramming by the S-nitroso-CoA reductase system protects against kidney injury. Nature 2019;565(7737):96-100.
